# Association of Computer-Assisted Virtual Preoperative Planning With Postoperative Mortality and Complications in Older Patients With Intertrochanteric Hip Fracture

**DOI:** 10.1001/jamanetworkopen.2020.5830

**Published:** 2020-08-10

**Authors:** Xiaoyang Jia, Kun Zhang, Minfei Qiang, Ying Wu, Yanxi Chen

**Affiliations:** 1 Department of Orthopedic Surgery, Zhongshan Hospital, Fudan University, Shanghai, China; 2 Department of Orthopedic Trauma, East Hospital, Tongji University School of Medicine, Shanghai, China; 3 Department of Biostatistics, School of Public Health, Guangdong Provincial Key Laboratory of Tropical Disease Research, Southern Medical University, Guangdong, Guangzhou, China

## Abstract

**Importance:**

The outcomes of surgical treatment in patients with intertrochanteric hip fractures are unsatisfactory. Computer-assisted virtual preoperative planning may provide an opportunity to solve this treatment dilemma. Virtual preoperative planning is a technique based on dynamic 3-dimensional computed tomographic imaging, which allows precise evaluation of fracture details and simulation of reduction of fracture and internal fixation procedures before surgery is performed.

**Objective:**

To evaluate the association of computer-assisted virtual preoperative planning with the risk of 90-day all-cause mortality and postoperative complications.

**Design, Setting, and Participants:**

This retrospective cohort study was conducted from using patient data from a level 1 trauma center database. A total of 1445 patients 65 years and older with intertrochanteric hip fractures between January 1, 2009, and March 31, 2018, were identified and divided into 2 cohorts: 558 patients received computer-assisted virtual preoperative planning (virtual planning group), and 887 patients received conventional preoperative planning (conventional planning group). Of the initial 1445 patients, 224 patients (93 patients in the virtual planning group and 131 patients in the conventional planning group) were excluded, resulting in 1221 patients in the final cohort. Data were analyzed from April 5 to October 5, 2019.

**Exposures:**

Computer-assisted virtual vs conventional surgical preoperative planning.

**Main Outcomes and Measures:**

Primary outcomes were 90-day all-cause mortality and postoperative complications (including myocardial infarction, heart failure, stroke, kidney failure, and sepsis). Secondary outcomes were 90-day outpatient visits, hospital readmissions, and reoperations.

**Results:**

Among 1221 patients who underwent hip surgery, the mean (SD) age was 73.2 (12.3) years, and 927 patients (75.9%) were women. A total of 465 patients (38.1%) were in the virtual planning group and 756 patients (61.9%) were in the conventional planning group. Among the 814 patients (407 patients in each group) who were matched by propensity score, the virtual planning group had a lower incidence of mortality (37 patients [9.1%] vs 55 patients [13.5%]; hazard ratio [HR], 0.64; 95% CI, 0.41-0.99; *P* = .04) and postoperative complications (25 patients [6.1%] vs 44 patients [10.8%]; HR, 0.54; 95% CI, 0.32-0.90; *P* = .02) compared with the conventional planning group. The incidence of outpatient visits was not substantially different in the virtual planning group (1.51 incidents per 30 person-days) compared with the conventional planning group (1.48 incidents per 30 person-days; incidence rate ratio [IRR], 0.90; 95% CI, 0.49-1.68; *P* = .75). Similar results were observed for the rate of hospital readmissions (0.99 incidents per 30 person-days in the virtual planning group and 1.01 incidents per 30 person-days in the conventional planning group; IRR, 0.91; 95% CI, 0.49-1.67; *P* = .76). However, the rate of reoperations was lower in the virtual planning group (0.76 incidents per 30 person-days) than in the conventional planning group (0.97 incidents per 30 person-days; IRR, 0.41; 95% CI, 0.22-0.76; *P* = .01).

**Conclusions and Relevance:**

Among older patients with intertrochanteric hip fractures, computer-assisted virtual preoperative planning was associated with decreases in the risks of all-cause 90-day mortality, postoperative complications, and reoperations compared with conventional preoperative planning.

## Key Points

**Question:**

Is preoperative planning using computer-assisted virtual surgical technology associated with decreases in the risks of all-cause 90-day mortality and postoperative complications among older patients with intertrochanteric hip fractures?

**Findings:**

In this cohort study of 1221 older patients with intertrochanteric hip fractures, the use of computer-assisted virtual preoperative planning among a propensity score–matched cohort was associated with a lower incidence of 90-day mortality and postoperative complications compared with the use of conventional preoperative planning.

**Meaning:**

This study’s findings suggest that preoperative planning using computer-assisted virtual surgical technology for older patients with intertrochanteric hip fractures is associated with decreases in the risks of all-cause 90-day mortality and postoperative complications.

## Teaser

This cohort study uses data from a level 1 trauma center database to evaluate the association of computer-assisted virtual preoperative planning with the risk of 90-day all-cause mortality and postoperative complications among older patients with intertrochanteric hip fractures in China.

## Introduction

Hip fractures are the most common type of fracture in patients older than 50 years.^1,2^ More than 300 000 hip fractures occur in the US each year.^2,3^ With the aging of the population, it is estimated that by 2040, the annual number of hip fractures will double.^3,4^ It has been reported that nearly one-half of hip fractures occur in the intertrochanteric region,^4^ and the outcomes of intertrochanteric hip fractures are worse if the fractures are left untreated. Therefore, early surgical intervention in patients with intertrochanteric fractures remains the standard of treatment. However, the postoperative outcomes are unsatisfactory owing to a higher risk of mortality, functional worsening, and diminished quality of life, which may impose a burden on health care systems.^5,6^ Unfortunately, most risk factors (including age, sex, and comorbidities) associated with worse postoperative outcomes in patients with intertrochanteric fractures are not modifiable.^7^

The unsatisfactory postoperative outcomes for patients with intertrochanteric fractures represent an opportunity to improve the treatment of this injury, and effective preoperative planning may provide a means to do so. Conventional preoperative planning has been performed using a combination of imaging data and the surgeon’s experience, which is the standard method used by most orthopedic surgeons in clinical settings. However, the outcomes of conventional preoperative planning for the treatment of intertrochanteric fractures have been unsatisfactory.^5,6,7,8,9,10^ In the past 20 years, a more advanced method of preoperative planning, computer-assisted virtual surgical technology based on computed tomographic postprocessing, has been used in the surgical management of some fractures, and clinical outcomes have improved to a certain extent.^11,12,13^ The benefits observed in preoperative planning using virtual surgical technology may be owing to the fact that this approach allows surgeons to observe fracture characteristics, such as the direction of the fracture line, the size of the fracture, and the number and location of fragments. In addition, surgeons can perform the operation virtually^11,14^

To our knowledge, no data exist to recommend 1 type of preoperative planning over another for the treatment of intertrochanteric fractures. Therefore, the aim of this retrospective cohort study was to investigate whether preoperative planning using computer-assisted virtual surgical technology was associated with decreases in the risks of mortality and postoperative complications and improvements in postoperative functional outcomes. A secondary aim was to examine whether the junior surgical residents, through the use of virtual surgical planning, would experience a faster learning curve regarding the treatment of these fractures.

## Methods

### Data Sources and Study Population

This study was a retrospective cohort analysis using information obtained from the electronic medical record database of East Hospital, Tongji University School of Medicine, a level 1 trauma center in Shanghai, China. The database contains patient data that include population characteristics at hospital admission, injury details, and surgical notes. The postoperative data of patients were obtained from the follow-up records. The study was approved by the institutional review board of East Hospital, Tongji University School of Medicine, with a waiver of informed consent because all data were deidentified. This study followed the Strengthening the Reporting of Observational Studies in Epidemiology (STROBE) reporting guideline for cohort studies.

We identified all patients 65 years and older on the date of hospital admission who had an intertrochanteric hip fracture and were treated with the proximal femoral nail antirotation 2 (PFNA-II, which has a smaller medial lateral angle and is more appropriately designed for the Asian population compared with the original PFNA) technique between January 1, 2009, and March 31, 2018. Patients who had multiple traumatic injuries, bilateral intertrochanteric hip fractures, pathological fractures, fractures that occurred during an inpatient hospital stay, previous fractures or surgery performed on the currently fractured site, and patients who transferred to another hospital after surgery or discharged from the hospital against medical advice were excluded.

A total of 1445 patients who underwent surgery for intertrochanteric hip fractures were identified. Of those, 224 patients (93 patients in the virtual planning group and 131 patients in the conventional planning group) were excluded because they met 1 or more of the following exclusion criteria: follow-up period of less than 1 year (126 patients [56.3%]), repeated fracture or surgery on the currently fractured site (42 patients [18.8%]), and/or nonadherence to postoperative rehabilitation guidance (56 patients [25.0%]). The final study cohort of 1221 patients included 465 patients (38.1%) who received computer-assisted virtual preoperative planning (virtual planning group) and 756 patients (61.9%) who received conventional preoperative planning (conventional planning group).

### Exposure Variable

**Figure 1.**
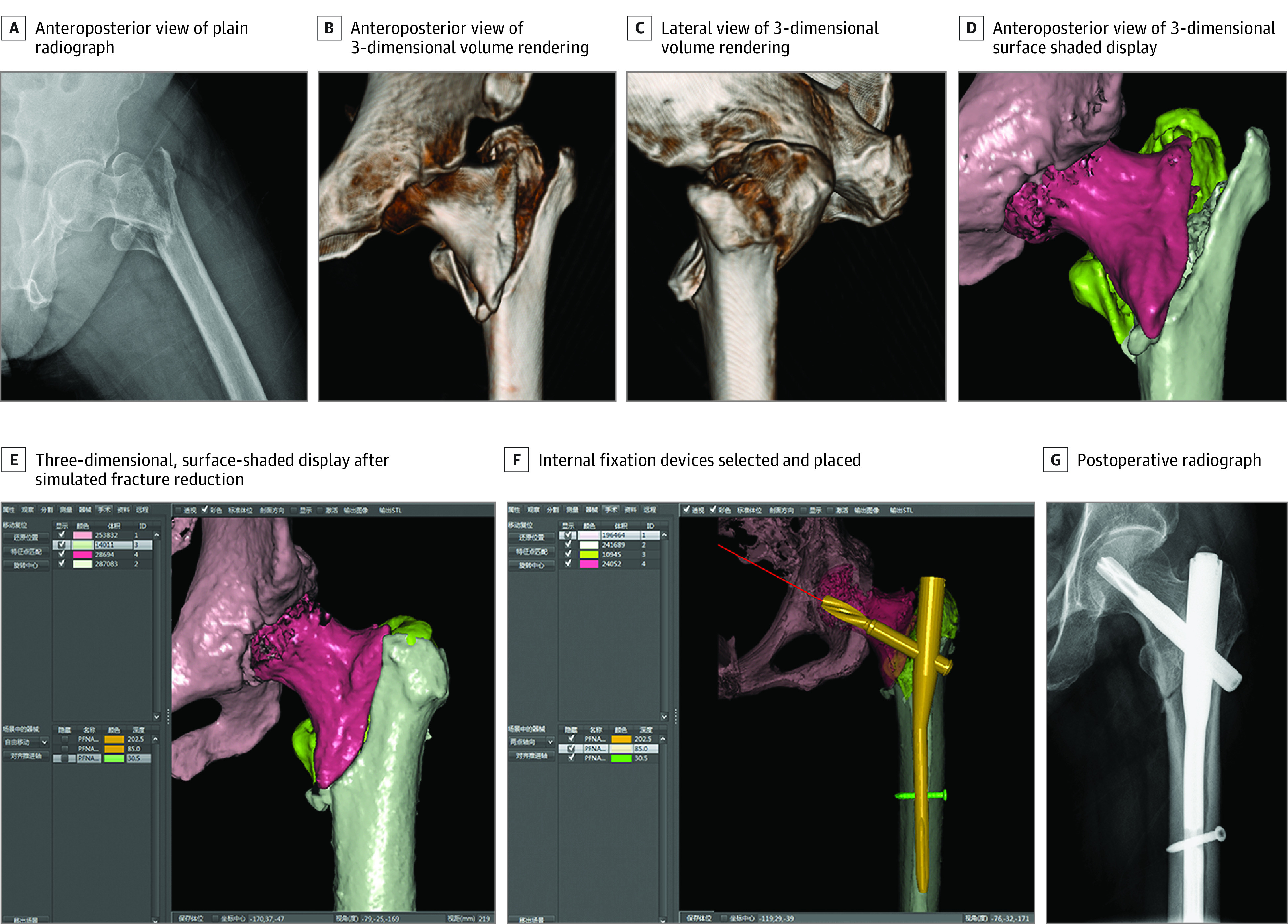
Application of Computer-Assisted Virtual Surgical Technology for Preoperative Planning in a Patient With Intertrochanteric Hip Fracture The image is from a woman aged 65 years who sustained an intertrochanteric hip fracture after falling to the floor (AO Foundation/Orthopaedic Trauma Association classification 31-A2, indicating comminuted fracture involving the lateral cortex). This image represents the consistency between the surgical procedure and preoperative planning using computer-assisted virtual surgical technology.

The exposure was the type of preoperative planning received by patients with intertrochanteric fractures, which comprised either planning using conventional methods or planning using computer-assisted virtual surgical technology. In the conventional planning group, preoperative planning was based on plain radiographic images and computed tomographic scans, including 2-dimensional and/or 3-dimensional (3-D) volume-rendering imaging, of the injured limb along with the surgeon’s experience, which is the standard method used by most orthopedic surgeons. In the virtual planning group, computed tomographic scans, which were obtained using a 16-detector spiral scanner (GE LightSpeed 16; GE Medical Systems), were entered into a computer-assisted orthopedic clinical research platform (SuperImage system, orthopedic edition 1.1; Cybermed).^11,15^ A 3-D image of the fracture in the proximal femur was reconstructed using a surface-shaded display algorithm, and fracture fragments were marked with distinct colors (Figure 1). The reduction of fracture procedure was simulated (Figure 1), and the suitable size of intramedullary devices in the PFNA-II system (including the length and diameter of the main nail, spiral blade, and distal locking screw) was chosen (Figure 1).

The surgical procedures were performed by our surgery team, which included 2 junior surgical residents (who began performing operations for intertrochanteric fractures in January 2009) and several senior surgeons (with more than 20 years of experience in performing operations for intertrochanteric fractures, including Y.C.). When a junior surgical resident performed the surgery, the operation was closely supervised by faculty. All surgical procedures were performed in accordance with the standard process and preoperative planning method.

### Outcomes

The primary outcomes were all-cause mortality and postoperative complications (including myocardial infarction, heart failure, stroke, kidney failure, and sepsis) within 90 days after the surgery. The primary outcomes were identified based on outpatient and/or telephone records. The secondary outcome was use of the health care system owing to postoperative complications, which was measured by the incidence rates of outpatient visits, hospital readmissions, and reoperations within 90 days after surgery. Patients with multiple reoperations and readmissions were counted only once. Moreover, only readmissions associated with other reasons (eg, other medical conditions) were counted, and those associated with reoperations were excluded.

Functional outcomes were evaluated at the 12-month follow-up using the Harris hip score,^16^ the Short Form 36 Health Survey Physical Component Summary (SF-36 PCS) scoring system, and the visual analog scale (VAS) for pain. The Harris hip score is used to assess the results of hip surgery and provides a numerical rating of hip function (score range, 0-100 points, with 0-69 indicating poor function, 70-79 indicating fair function, 80-89 indicating good function, and 90-100 indicating excellent function). The SF-36 is a 36-item patient-reported survey of patient health (comprising 8 scaled scores, which are the weighted sums of the questions in their sections); the SF-36 PCS outcome scale was used in this study, with each score normalized to a 100-point scale (calculated as the raw score minus 10 multiplied by 100 divided by 20, with higher scores indicating less disability). The VAS is used to measure the intensity of pain using a numerical rating scale (score range, 0-10 points, with 0 indicating no pain and 10 indicating maximum pain).

### Learning Curve

Patients who received treatment from junior surgical residents and who had data available regarding the duration of the closed reduction procedure, the duration of surgery, the number of fluoroscopic images performed, the estimated amount of blood loss, and the conversion rates to open reduction procedures were used to evaluate the learning curve. The duration of the closed reduction of fracture procedure was recorded as the time from the beginning of the reduction procedure to disinfection. The duration of surgery was recorded as the time from skin incision to closure.

The number of fluoroscopic images performed was recorded as the count of fluoroscopic images taken from the beginning of the reduction procedure to the end of the surgery. The estimated amount of blood loss was recorded as the amount of bleeding (measured in mL) that occurred during the surgery. For complex intertrochanteric hip fractures in which it was difficult to insert the guide pin during surgery, open reduction and internal fixation procedures were performed. Therefore, the conversion rates to open reduction procedures were also recorded to analyze the learning curve.

### Statistical Analysis

To balance the potential differences in baseline characteristics between the 2 groups, propensity score matching was performed. The propensity score was estimated using logistic regression analysis based on the patient’s sociodemographic characteristics, functional status before injury, mechanism of injury, affected side of hip, fracture classification, timing of surgery, medical history, year of surgery, and status according to the American Society of Anesthesiologists (ASA) Physical Status Classification System (which assesses a patient’s medical comorbidities before anesthesia; status range, ASA 1 to ASA 6, with ASA 1 indicating a healthy patient with no disease, ASA 2 indicating a patient with mild systemic disease, ASA 3 indicating a patient with severe systemic disease, ASA 4 indicating a patient with severe systemic disease that is life-threatening, ASA 5 indicating a patient who is not expected to survive without surgery, and ASA 6 indicating a patient in whom brain death has occurred; higher ASA status indicates greater risk during anesthesia). One-to-one nearest-neighbor caliper matching was used to match patients based on the logit function of the propensity score using a caliper equal to 0.02 of the SD of the logit function of the propensity score.^17^ Patients were exactly matched according to the baseline timing of surgery.

For a given covariate, a standardized difference of less than 10% indicates a relatively small imbalance.^18^ The balance of baseline characteristics was also assessed in patients with and without early surgery (≤24 hours after injury and >24 hours after injury, respectively) because the comparisons between patients in the virtual planning group and the conventional planning group were stratified by the occurrence of early surgery.

A Cox proportional hazards regression analysis was used to compare patients in the virtual planning group with those in the conventional planning group for all-cause 90-day mortality in the propensity score–matched cohort, with robust sandwich estimates used to account for the clustering within matched sets.^19^ For the end point of 90-day postoperative complications, a competing risk analysis was performed to construct cumulative incidence function curves, and the difference between the 2 groups was evaluated using the Fine and Gray test.^20^ The proportional hazards assumption was examined using the Schoenfeld residuals test.^21^ Poisson regression analysis was used to assess complication-associated outpatient visits, reoperations, and readmissions. Subgroup analyses for 90-day mortality and postoperative complications were stratified by age, sex, functional status before injury, ASA status, AO Foundation/Orthopaedic Trauma Association fracture classification (classification range, A1 to A3, with A1 indicating simple fracture, A2 indicating comminuted fracture involving the lateral cortex, and A3 indicating reverse oblique fracture), and the presence of anemia, atrial fibrillation, chronic kidney disease, heart failure, intracranial bleeding, and myocardial infarction. The consistency of hazard ratios (HRs) across subgroups was tested by the significance of the interaction terms, and the subgroup analyses were regarded as exploratory.

The baseline characteristics of patients are potential confounding factors for assessing postoperative functional outcomes. Therefore, a multivariable linear regression analysis was performed to compare the differences in functional outcomes (including the Harris hip score, the SF-36 PCS score, and the VAS score) between the 2 groups. Outcomes of the learning curve were reviewed in groups of 25 cases to minimize the consequences of demographic differences and to optimize normalcy in distribution. To account for confounding factors, a multivariable linear regression analysis was used to evaluate the changes in the learning curve for the duration of the closed reduction procedure, the duration of surgery, the number of fluoroscopic images performed, and the estimated amount of blood loss, and a logistic regression analysis was performed for the conversion rate to open reduction procedures. Baseline variables that were considered clinically relevant or that had a *P* value less than .10 on the univariate analysis were entered into the regression model. Variables for inclusion were carefully chosen, given the number of incidents available, to ensure parsimony of the final model. All tests were paired and 2-sided, with a significance threshold of *P* < .05. All statistical analyses were performed using Stata software, version 14.1 (StataCorp LLC) and SAS software, version 9.4 (SAS Institute Inc). Data were analyzed from April 5 to October 5, 2019.

## Results

### Study Population

Among 1221 patients who underwent hip surgery, the mean (SD) age was 73.2 (12.3) years; 927 patients (75.9%) were women, 506 patients (41.4%) had an ASA status of 3 or higher, and 689 patients (56.4%) underwent early surgery (ie, surgery ≤24 hours after injury). Variables that differed between patients in the virtual planning group and the conventional planning group included sex, body mass index (calculated as weight in kilograms divided by height in meters squared), and educational level. Patients in the virtual planning group (n = 465 [38.1%]) compared with those in the conventional planning group (n = 756 [61.9%]) were more likely to have a history of alcohol use (70 patients [15.1%] vs 76 patients [10.1%], respectively), depression (37 patients [8.0%] vs 30 patients [4.0%]), hypertension (348 patients [74.8%] vs 527 patients [69.7%]), and smoking (126 patients [27.1%] vs 158 patients [20.9%]) and were less likely to have a history of anemia (45 patients [9.7%] vs 113 patients [14.9%]), atrial fibrillation (18 patients [3.9%] vs 54 patients [7.1%]), diabetes (83 patients [17.8%] vs 166 patients [22.0%]), heart failure (23 patients [4.9%] vs 61 patients [8.1%]), and myocardial infarction (34 patients [7.3%] vs 90 patients [11.9%]) (Table 1).

**Table 1.** Baseline Demographic Characteristics of Patients Before and After Propensity Score Matching Based on Type of Preoperative Planning

Characteristic	Before propensity score matching			After propensity score matching		
	No. (%)		Standardized difference, %	No. (%)		Standardized difference, %
	Virtual planning (n = 465)	Conventional planning (n = 756)		Virtual planning (n = 407)	Conventional planning (n = 407)	
Age range, y						
65-69	64 (13.8)	87 (11.5)	6.9	53 (13.0)	47 (11.5)	4.3
70-74	175 (37.6)	322 (42.6)	10.2	158 (38.8)	176 (43.2)	8.7
75-79	151 (32.5)	220 (29.1)	7.4	130 (31.9)	118 (29.0)	6.3
≥80	75 (16.1)	127 (16.8)	1.9	66 (16.2)	66 (16.2)	0
Women	315 (67.7)	612 (81.0)	30.8	301 (74.0)	294 (72.2)	4.0
BMI range						
≤18.4	28 (6.0)	67 (8.9)	10.7	25 (6.1)	32 (7.9)	7.1
18.5-23.9	316 (68.0)	433 (57.3)	22.3	271 (66.6)	252 (61.9)	9.8
24.0-27.9	70 (15.1)	151 (20.0)	13.2	63 (15.5)	71 (17.4)	5.1
≥28.0	51 (11.0)	105 (13.9)	8.8	48 (11.8)	52 (12.8)	3.0
Educational level						
Primary school	295 (63.4)	381 (50.4)	26.5	253 (62.2)	264 (64.9)	5.5
Junior high school	68 (14.6)	265 (35.1)	48.8	57 (14.0)	56 (13.8)	0.7
High school or higher	102 (21.9)	110 (14.5)	19.5	97 (23.8)	87 (21.4)	5.8
Functional status before injury						
Independent	334 (71.8)	589 (77.9)	14.1	298 (73.2)	309 (75.9)	6.2
Partially dependent	123 (26.5)	154 (20.4)	14.4	102 (25.1)	89 (21.9)	7.6
Dependent	8 (1.7)	13 (1.7)	0	7 (1.7)	9 (2.2)	3.6
Injury mechanism						
Fell from height	331 (71.2)	575 (76.1)	11.1	293 (72.0)	308 (75.7)	8.4
Traffic accident	92 (19.8)	103 (13.6)	16.7	81 (19.9)	74 (18.2)	4.6
Other	42 (9.0)	78 (10.3)	4.4	33 (8.1)	25 (6.1)	7.3
Affected side						
Left	265 (57.0)	491 (64.9)	16.2	242 (59.5)	238 (58.5)	2.0
Right	200 (43.0)	265 (35.1)	16.2	165 (40.5)	169 (41.5)	2.0
ASA status^a^						
1-2	269 (57.8)	446 (59.0)	2.4	236 (58.0)	247 (60.7)	5.5
3	168 (36.1)	250 (33.1)	6.3	146 (35.9)	129 (31.7)	8.9
4	28 (6.0)	60 (7.9)	7.1	25 (6.1)	31 (7.6)	5.9
AO/OTA classification^b^						
A1	277 (59.6)	476 (63.0)	7.0	243 (59.7)	247 (60.7)	2.0
A2	158 (34.0)	243 (32.1)	4.0	139 (34.2)	135 (33.2)	2.1
A3	30 (6.5)	37 (4.9)	6.5	25 (6.1)	25 (6.1)	0
Timing of surgery after injury, h						
≤24	251 (54.0)	438 (57.9)	7.9	220 (54.1)	220 (54.1)	0
>24	214 (46.0)	318 (42.1)	7.9	187 (45.9)	187 (45.9)	0
Medical history						
Alcohol use	70 (15.1)	76 (10.1)	15.4	50 (12.3)	54 (13.3)	3.3
Anemia	45 (9.7)	113 (14.9)	15.9	41 (10.1)	48 (11.8)	5.4
Atrial fibrillation	18 (3.9)	54 (7.1)	14.1	17 (4.2)	22 (5.4)	5.6
Cancer	23 (4.9)	45 (6.0)	4.8	19 (4.7)	23 (5.7)	4.5
Cancer with metastasis	10 (2.2)	15 (2.0)	1.2	7 (1.7)	9 (2.2)	1.5
Chronic kidney disease	33 (7.1)	38 (5.0)	8.8	24 (5.9)	23 (5.7)	0.9
COPD	40 (8.6)	85 (11.2)	8.7	40 (9.8)	37 (9.1)	2.4
Dementia	5 (1.1)	13 (1.7)	5.1	4 (1.0)	3 (0.7)	2.7
Depression	37 (8.0)	30 (4.0)	16.9	25 (6.1)	27 (6.6)	2.1
Diabetes	83 (17.8)	166 (22.0)	10.3	75 (18.4)	81 (19.9)	3.8
Heart failure	23 (4.9)	61 (8.1)	12.7	21 (5.2)	24 (5.9)	3.0
Hypertension	348 (74.8)	527 (69.7)	11.5	302 (74.2)	297 (73.0)	2.8
Intracranial bleeding	4 (0.9)	12 (1.6)	6.6	4 (1.0)	3 (0.7)	2.7
Liver disease	48 (10.3)	99 (13.1)	8.7	46 (11.3)	44 (10.8)	6.2
Myocardial infarction	34 (7.3)	90 (11.9)	15.6	32 (7.9)	32 (7.9)	0
Smoking	126 (27.1)	158 (20.9)	14.6	100 (24.6)	96 (23.6)	2.3
Year of surgery						
2009	43 (9.2)	64 (8.5)	2.8	40 (9.8)	37 (9.1)	2.4
2010	51 (11.0)	75 (9.9)	3.6	40 (9.8)	48 (11.8)	6.4
2011	55 (11.8)	76 (10.1)	5.4	45 (11.1)	52 (12.8)	5.2
2012	54 (11.6)	85 (11.2)	1.3	47 (11.5)	49 (12.0)	1.6
2013	50 (10.8)	87 (11.5)	2.2	47 (11.5)	47 (11.5)	0
2014	55 (11.8)	85 (11.2)	1.9	47 (11.5)	45 (11.1)	1.3
2015	61 (13.1)	86 (11.4)	5.2	54 (13.3)	47 (11.5)	5.2
2016	41 (8.8)	84 (11.1)	7.7	34 (8.4)	32 (7.9)	1.8
2017	43 (9.2)	85 (11.2)	6.6	41 (10.1)	36 (8.8)	4.1
2018	12 (2.6)	29 (3.8)	7.3	12 (2.9)	14 (3.4)	2.3

Abbreviations: AO/OTA, AO Foundation/Orthopaedic Trauma Association; ASA, American Society of Anesthesiologists; BMI, body mass index (calculated as weight in kilograms divided by height in meters squared); COPD, chronic obstructive pulmonary disease.

^a^ ASA Physical Status Classification System status range, ASA 1 to ASA 6 (with ASA 1 indicating a healthy patient with no disease, ASA 2 indicating a patient with mild systemic disease, ASA 3 indicating a patient with severe systemic disease, ASA 4 indicating a patient with severe systemic disease that is life-threatening, ASA 5 indicating a patient who is not expected to survive without surgery, and ASA 6 indicating a patient in whom brain death has occurred). Higher ASA status indicates greater risk during anesthesia.

^b^ AO/OTA fracture classification range, A1 to A3, representing different types of fractures (with A1 indicating simple fracture, A2 indicating comminuted fracture involving the lateral cortex, and A3 indicating reverse oblique fracture).

Propensity score matching produced 407 patient pairs. All baseline characteristics were balanced between the 2 groups (Table 1), and standardized differences in baseline characteristics did not exceed 10%. Of the 814 patients matched by propensity score, 440 patients (54.1%) underwent early surgery. Baseline characteristics stratified by early surgery are reported in eTable 1 and eTable 2 in the Supplement. In patients who did and did not receive early surgery, the baseline characteristics were balanced between those in the virtual planning group and those in the conventional planning group.

### Primary and Secondary Outcomes

Among 814 patients (407 patients in each group) matched by propensity score, the virtual planning group experienced a lower incidence of 90-day mortality (37 patients [9.1%]; 95% CI, 6.4%-12.5% vs 55 patients [13.5%]; 95% CI, 10.2%-17.6%; HR, 0.64; 95% CI, 0.41-0.99; *P* = .04) and postoperative complications (25 patients [6.1%]; 95% CI, 4.0%-9.1% vs 44 patients [10.8%]; 95% CI, 7.9%-14.5%; HR, 0.54; 95% CI, 0.32-0.90; *P* = .02) than the conventional planning group (Table 2; eFigure 1 and eFigure 2 in the Supplement).

**Table 2.** All-Cause Mortality and Postoperative Complications Within 90 Days After Surgery in Patients Matched by Propensity Score

Outcome	Virtual planning			Conventional planning			HR (95% CI)	*P* value
	Incidents, No./No. of patients (%)	Person-days, No.	Incidence rate^a^	Incidents, No./No. of patients (%)	Person-days, No.	Incidence rate^a^		
Death								
Overall	37/407 (9.1)	1606	0.69	55/407 (13.5)	1716	0.96	0.64 (0.41-0.99)	.04
Surgery ≤24 h after injury	16/220 (7.3)	755	0.64	29/220 (13.2)	890	0.98	0.52 (0.27-0.98)	.04
Surgery >24 h after injury	21/187 (11.2)	851	0.74	26/187 (13.9)	826	0.94	0.78 (0.42-1.45)	.44
Complications								
Overall	25/407 (6.1)	1177	0.64	44/407 (10.8)	1542	0.86	0.54 (0.32-0.90)	.02
Surgery ≤24 h after injury	12/220 (5.5)	584	0.62	28/220 (12.7)	876	0.96	0.40 (0.20-0.80)	.01
Surgery >24 h after injury	13/187 (7.0)	593	0.66	16/187 (8.6)	666	0.72	0.80 (0.37-1.71)	.56

Abbreviation: HR, hazard ratio.

^a^ Calculated as the number of incidents per 30 person-days.

The incidence of complication-associated outpatient visits within 90 days after surgery was not substantially different between the virtual planning group (1.51 incidents per 30 person-days) and the conventional planning group (1.48 incidents per 30 person-days; incidence rate ratio [IRR], 0.90; 95% CI, 0.49-1.68; *P* = .75). Similar results were observed for complication-associated hospital readmissions (0.99 incidents per 30 person-days in the virtual planning group and 1.01 incidents per 30 person-days in the conventional planning group; IRR, 0.91; 95% CI, 0.49-1.67; *P* = .76). However, the incidence of reoperations was lower in the virtual planning group (0.76 incidents per 30 person-days) than in the conventional planning group (0.97 incidents per 30 person-days; IRR, 0.41; 95% CI, 0.22-0.76; *P* = .01) (Table 3).

**Table 3.** Unplanned Outpatients Visits, Hospital Readmissions, and Reoperations Within 90 Days After Hospital Discharge in Patients Matched by Propensity Score

Outcome	Virtual planning			Conventional planning			IRR (95% CI)	*P* value
	Incidents, No./No. of patients (%)	Person-days, No.	Incidence rate^a^	Incidents, No./No. of patients (%)	Person-days, No.	Incidence rate^a^		
Outpatients visit								
Overall	20/407 (4.9)	398	1.51	22/407 (5.4)	445	1.48	0.90 (0.49-1.68)	.75
Surgery ≤24 h after injury	8/220 (3.6)	154	1.56	9/220 (4.1)	173	1.56	0.89 (0.34-2.34)	.81
Surgery >24 h after injury	12/187 (6.4)	244	1.48	13/187 (7.0)	272	1.43	0.92 (0.41-2.07)	.84
Hospital readmission								
Overall	21/407 (5.2)	636	0.99	23/407 (5.7)	683	1.01	0.91 (0.49-1.67)	.76
Surgery ≤24 h after injury	9/220 (4.1)	269	1.00	10/220 (4.5)	290	1.03	0.90 (0.36-2.25)	.82
Surgery >24 h after injury	12/187 (6.4)	367	0.98	13/187 (7.0)	393	0.99	0.92 (0.41-2.07)	.84
Reoperation								
Overall	15/407 (3.7)	592	0.76	35/407 (8.6)	1084	0.97	0.41 (0.22-0.76)	.01
Surgery ≤24 h after injury	7/220 (3.2)	268	0.78	17/220 (7.7)	531	0.96	0.39 (0.16-0.97)	.04
Surgery >24 h after injury	8/187 (4.3)	324	0.74	18/187 (9.6)	553	0.98	0.42 (0.18-0.99)	.04

Abbreviation: IRR, incidence rate ratio.

^a^ Calculated as the number of incidents per 30 person-days.

### Stratified Analyses and Functional Outcomes

Among the 440 patients in the matched cohort who underwent early surgery, the incidence of 90-day mortality was 16 of 220 patients (7.3%; 95% CI, 4.2%-11.8%) in the virtual planning group vs 29 of 220 patients (13.2%; 95% CI, 8.8%-18.9%) in the conventional planning group (HR, 0.52; 95% CI, 0.27-0.98; *P* = .04). The incidence of postoperative complications was 12 of 220 patients (5.5%; 95% CI, 2.8%-9.5%) in the virtual planning group vs 28 of 220 patients (12.7%; 95% CI, 8.5%-18.4%) in the conventional planning group (HR, 0.40; 95% CI, 0.20-0.80; *P* = .01). Among the 374 patients who did not receive early surgery (ie, surgery >24 hours after injury), the incidence of 90-day mortality was 21 of 187 patients (11.2%; 95% CI, 7.0%-17.2%) in the virtual planning group vs 26 of 187 patients (13.9%; 95% CI, 9.1%-20.4%) in the conventional planning group (HR, 0.78; 95% CI, 0.42-1.45; *P* = .44). The incidence of postoperative complications was 13 of 187 patients (7.0%; 95% CI, 3.7%-11.9%) in the virtual planning group vs 16 of 187 patients (8.6%; 95% CI, 4.9%-13.9%) in the conventional planning group (HR, 0.80; 95% CI, 0.37-1.71; *P* = .56) (Table 2; eFigure 1 and eFigure 2 in the Supplement).

Among patients matched by propensity score who had early surgery, the incidence rate of complication-associated outpatient visits was the same between the virtual planning group and the conventional planning group (1.56 incidents per 30 person-days; IRR, 0.89; 95% CI, 0.34-2.34; *P* = .81). For complication-associated hospital readmissions, the rate was 1.00 incident per 30 person-days in the virtual planning group vs 1.03 incidents per 30 person-days in the conventional planning group (IRR, 0.90; 95% CI, 0.36-2.25; *P* = .82). For complication-associated reoperations, the rate was 0.78 incidents per 30 person-days in the virtual planning group vs 0.96 incidents per 30 person-days in the conventional planning group (IRR, 0.39; 95% CI, 0.16-0.97; *P* = .04). Among patients who did not receive early surgery, the rate for outpatient visits was 1.48 incidents per 30 person-days in the virtual planning group vs 1.43 incidents per 30 person-days in the conventional planning group (IRR, 0.92; 95% CI, 0.41-2.07; *P* = .84). The rate for hospital readmissions was 0.98 incidents per 30 person-days in the virtual planning group vs 0.99 incidents per 30 person-days in the conventional planning group (IRR, 0.92; 95% CI, 0.41-2.07; *P* = .84). The rate for reoperations was 0.74 incidents per 30 person-days in the virtual planning group vs 0.98 incidents per 30 person-days in the conventional planning group (IRR, 0.42; 95% CI, 0.18-0.99; *P* = .04) (Table 3). The subgroup analyses were consistent with the findings of the main analyses (eTable 3, eTable 4, eTable 5, and eTable 6 in the Supplement).

Of the 1221 total patients, 195 patients (16.0%; 75 patients in the virtual planning group and 120 patients in the conventional planning group) died during the 12-month follow-up period. Among the remaining patients in the unmatched cohort (390 patients in the virtual planning group and 636 patients in the conventional planning group), the mean (SD) Harris hip score was 66.8 (9.6) points in the virtual planning group vs 66.1 (6.8) points in the conventional planning group (*P* = .16). The mean (SD) SF-36 PCS score was 70.3 (6.5) points in the virtual planning group vs 69.9 (5.8) points in the conventional planning group (*P* = .29). The mean (SD) VAS score was 3.5 (1.3) points in the virtual planning group vs 3.6 (1.2) points in the conventional planning group (*P* = .20) (eTable 7 in the Supplement). Similar results were observed in patients stratified by timing of surgery (with or without early surgery) with regard to the Harris hip score, the SF-36 PCS score, and the VAS score when comparative analyses were performed between patients in the virtual planning group and the conventional planning group (eTable 7 and eTable 8 in the Supplement).

### Learning Curve

**Figure 2.**
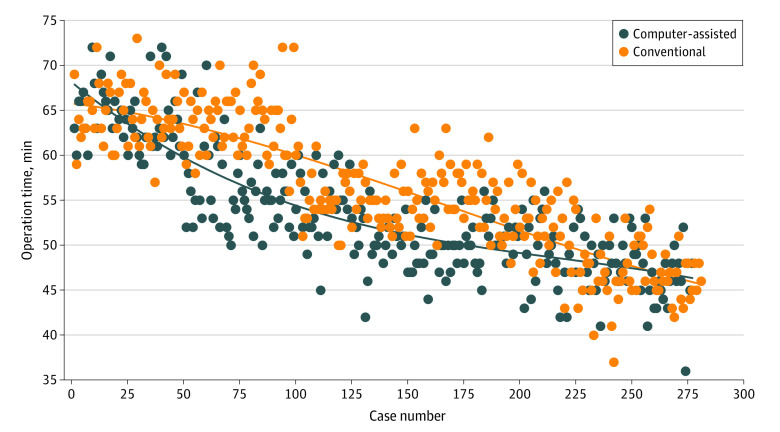
Learning Curves for Surgery Duration Using Computer-Assisted Virtual Preoperative Planning vs Conventional Preoperative Planning Virtual planning, *y* = −0.000001*x*^3^ + 0.0008*x*^2^ − 0.2023x + 68.082; *R*^2^ = 0.7155; *P* < .001. Conventional planning, *y* = 7*E* – 07*x*^3^ – 0.0003*x*^2^ – 0.0303*x* + 65.762; *R*^2^ = 0.7322; *P* < .001.

A total of 277 patients in the virtual planning group and 281 patients in the conventional planning group received treatment from junior surgical residents. Patient demographic characteristics were evaluated to monitor selection bias (eTable 9 and eTable 10 in the Supplement). Significant improvements in the duration of surgery were observed after 50 cases (mean [SD], 64.2 [3.5] minutes for cases 1-50 vs 52.2 [4.8] minutes for cases 51-277; *P* < .001) in the virtual planning group and after 100 cases (mean [SD], 63.1 [3.5] minutes for cases 1-100 vs 51.9 [4.9] minutes for cases 101-281; *P* < .001) in the conventional planning group. In the conventional planning group, an incremental but statistically significant decrease in the duration of surgery was also observed between cases 51 through 125 and cases 126 through 277 (mean [SD], 55.8 [4.1] minutes vs 48.6 [3.4] minutes, respectively; *P* < .001) in the virtual planning group and between cases 101 through 200 and cases 201 through 281 (mean [SD], 55.5 [3.0] minutes vs 48.2 [4.1] minutes, respectively; *P* < .001) in the conventional planning group. Figure 2 shows the learning curves for surgery duration in the 2 groups (in the virtual planning group, *y* = −0.000001*x*^3^ + 0.0008*x*^2^ − 0.2023x + 68.082; *R*^2^ = 0.7155; *P* < .001 for cubic regression; in the conventional planning group, *y* = 7*E* – 07*x*^3^ – 0.0003*x*^2^ – 0.0303*x* + 65.762; *R*^2^ = 0.7322; *P* < .001 for cubic regression). The learning curve for surgery duration in the virtual planning group was faster and reached the platform stage (ie, the point at which the surgery duration could not be reduced further) earlier compared with that of the conventional planning group (Figure 2).

The duration of the closed reduction of fracture procedure indicated a significant improvement after 50 cases (mean [SD], 33.2 [3.3] minutes for cases 1-50 vs 23.0 [2.4] minutes for cases 51-277; *P* < .001) in the virtual planning group and after 75 cases (mean [SD], 35.0 [3.0] minutes for cases 1-75 vs 23.5 [2.7] minutes for cases 76-281; *P* < .001) in the conventional planning group. The number of fluoroscopic images performed and the estimated amount of blood loss indicated substantial decreases after 50 cases in the virtual planning group and 100 cases in the conventional planning group. The learning curve for the duration of the closed reduction procedure, the number of fluoroscopic images performed, and the estimated amount of blood loss among surgical residents performing operations in the virtual planning group was faster and reached the platform stage earlier compared with the learning curve of surgical residents performing operations in the conventional planning group (eFigure 3, eFigure 4, and eFigure 5 in the Supplement). A similar improvement in the conversion rate to open reduction procedures was observed after 26 cases in the virtual planning group (5 of 25 procedures [20.0%] for cases 1-25 vs 14 of 252 procedures [5.6%] for cases 26-277; *P* < .001) and 75 cases in the conventional planning group (15 of 75 procedures [20.0%] for cases 1-75 vs 16 of 206 procedures [8.0%] for cases 76-281; *P* < .001).

## Discussion

In this retrospective cohort study of patients with intertrochanteric hip fractures, the receipt of preoperative planning based on computer-assisted virtual surgical technology compared with conventional methods was associated with a lower risk of mortality, postoperative complications, and reoperations within 90 days after surgery. However, the benefits of virtual surgical technology were not observed for complication-associated outpatient visits, hospital readmissions, and functional outcomes. The learning curve for surgical residents who performed operations in the virtual planning group was faster than that of surgical residents who performed operations in the conventional planning group.

The benefits of computer-assisted virtual preoperative planning for mortality, postoperative complications, and reoperations may be associated with several factors. First, for complex intertrochanteric hip fractures, virtual planning allows surgeons to observe the characteristics and injury details of fracture fragments (such as fracture line orientation, size, and number and position of fragments) from multiple perspectives before performing surgery and to evaluate the possibility of reduction for each fragment. Second, by using a preoperative simulation of the reduction procedure, surgeons can assess the important anatomical landmarks of reduction and the integrity of the lateral femoral wall, identify the key fracture fragments that need to be restored, and evaluate whether a bone defect is present after the reduction procedure. Third, surgeons can simulate the implantation of internal fixation devices before performing surgery to obtain reliable information about the appropriate size of the device. The acquisition of this detailed information is conducive to achieving fracture reduction and implanting internal fixation devices rapidly and accurately during surgery. This information may also reduce the duration of intraoperative reduction procedures and the number of fluoroscopic images performed, which can decrease the duration of surgery and the amount of intraoperative blood loss and improve the success rate of reduction and fixation procedures.

The advantages observed in the virtual planning group may be associated with the faster learning curve for surgical resident using computer-assisted virtual surgical technology compared with conventional methods. In addition, the computer-assisted preoperative planning system is efficient and convenient. In this study, the mean duration of virtual preoperative planning was approximately 27.0 minutes. The computer-assisted preoperative planning was completed in a software system, in which surgeons could easily and accurately control the 3-D surface reconstruction image based on computed tomographic scanning data and could complete the free segmentation and free editing of the 3-D image of each fracture fragment (such as the movement and reduction of the fragment) in a few minutes. In addition, the appropriate internal fixation devices could be selected for virtual implantation.

The benefits observed in the virtual planning group were present among patients who received early surgery but not among those who received delayed surgery. A hip fracture is often associated with traumatic injury, pain, bleeding, and immobility. These factors initiate inflammatory, hypercoagulable, stress-associated, and catabolic states that have the potential to produce complications, such as myocardial infarction, pulmonary embolism, pneumonia, sepsis, stroke, major bleeding, disability, and mortality.^22,23^ It is possible to minimize a patient’s exposure to these harmful factors through early surgery.^22,24^ Therefore, the potential harms of delaying surgery may offset the benefits of preoperative planning using computer-assisted virtual surgical technology.

No significant differences were found between the virtual planning group and the conventional planning group with regard to outpatient visits, hospital readmissions, and functional outcomes. The function and status of patients discharging from the hospital were associated with the subsequent medical specialty and the location of postoperative care. Furthermore, the patient’s completion or noncompletion of postoperative rehabilitation, including timely mobilization, early rehabilitation, and postacute care, was likely associated with these outcomes. Therefore, the rates of outpatient visits, hospital readmissions, and functional outcomes were the balanced results of preoperative planning and the factors discussed in this paragraph.

### Limitations

This study has several limitations. First, given its retrospective observational design, there was the potential for selection bias and confounding bias. Therefore, propensity score matching and regression analyses were used to obtain well-balanced groups. Despite the performance of procedures to minimize selection and confounding biases, the possibility of residual confounding (ie, the presence of comorbid factors that were not measured by the data available for analysis) remained. The presence of unmeasured comorbidities in patients with intertrochanteric hip fractures may have had implications for the study’s outcomes. Thus, further research is needed through a larger randomized prospective study.

Second, with the exception of experience with intertrochanteric hip fractures, it was not clear whether differences existed in the experience of the 2 junior surgical residents and the senior surgeons with regard to other types of fractures. These differences may have had implications for their treatment of intertrochanteric hip fractures. However, in China, junior surgical residents are not permitted to perform operations without consultant supervision. Furthermore, the PFNA-II technique is generally less invasive than other methods and is a relatively simple surgery to perform. Therefore, even if differences existed between the 2 junior surgical residents and the senior surgeons regarding their experience with other types of fractures, the implications for our results may be minimal.

Third, we were not able to adjust our analyses for any medical circumstances that may have been factors in the postoperative outcomes of patients discharging from the hospital. However, patients who received treatment from our research team participated in standard rehabilitation programs during the follow-up period. Therefore, the medical circumstances at discharge for postoperative outcomes would have been controlled. Fourth, when evaluating the differences in postoperative functional outcomes between the 2 groups, death was considered the appropriate censoring point, which likely produced bias in the results. To evaluate the implications of this bias, the mean score of the group with the lower function score was used as the score of the patients who had died; thus, the data were supplemented. The data indicated that the results were consistent with those before supplementation, and no significant difference was observed.

## Conclusions

Among older patients with intertrochanteric hip fractures who received preoperative planning, the use of computer-assisted virtual surgical technology was associated with decreases in the risks of 90-day mortality, postoperative complications, and reoperations compared with the use of conventional methods. Virtual preoperative planning was also associated with a faster learning curve for junior surgical residents compared with conventional planning. However, the benefits of virtual planning were not found for outpatient visits, hospital readmissions, and functional outcomes. These findings support the use of preoperative planning based on computer-assisted virtual surgical technology for older patients with intertrochanteric fractures. Further studies are needed to define the role of virtual surgical technology.

## Notes

Supplement. **eTable 1.** Baseline Demographic Characteristics in Patients With Early Surgery Before and After Propensity Score Matching According to Type of Preoperative Planning **eTable 2.** Baseline Demographic Characteristics in Patients Without Early Surgery Before and After Propensity Score Matching According to Type of Preoperative Planning **eTable 3.** Subgroup Analysis for Death in Propensity Score–Matched Patients With Early Surgery **eTable 4.** Subgroup Analysis for Complications in Propensity Score–Matched Patients With Early Surgery **eTable 5.** Subgroup Analysis for Death in Propensity Score–Matched Patients Without Early Surgery **eTable 6.** Subgroup Analysis for Complications in Propensity Score–Matched Patients Without Early Surgery **eTable 7.** Postoperative Clinical Outcomes in 1-Year Follow-Up of Unmatched Patients **eTable 8.** Postoperative Clinical Outcomes in 1-Year Follow-Up of All Unmatched Patients **eTable 9.** Baseline Characteristics of 277 Consecutive Patients Treated by Junior Resident With Use of Computer-Assisted Preoperative Planning **eTable 10.** Baseline Characteristics of 281 Consecutive Patients Treated by Junior Resident With Use of Conventional Preoperative Planning **eFigure 1.** Risk of All-Cause 90-Day Mortality in Propensity Score–Matched Patients **eFigure 2.** Cumulative Incidence of All-Cause 90-Day Complications in Propensity Score–Matched Patients **eFigure 3.** Learning Curves of Closed Reduction of Fracture Time With Use of Computer-Assisted and Conventional Preoperative Planning **eFigure 4.** Learning Curves of Number of Fluoroscopies With Use of Computer-Assisted and Conventional Preoperative Planning **eFigure 5.** Learning Curves of Estimated Blood Loss With Use of Computer-Assisted and Conventional Preoperative Planning
