# Supplementary material for: Association of Computer-Assisted Virtual Preoperative Planning With Postoperative Mortality and Complications in Older Patients With Intertrochanteric Hip Fracture
Source: JAMA Netw Open. 2020 Aug 10;3(8):e205830. doi: 10.1001/jamanetworkopen.2020.5830 (PMC7417968; doi:10.1001/jamanetworkopen.2020.5830)
Supplement: Supplement. — eTable 1. Baseline Demographic Characteristics in Patients With Early Surgery Before and After Propensity Score Matching According to Type of Preoperative Planning eTable 2. Baseline Demographic Characteristics in Patients Without Early Surgery Before and After Propensity Score Matching According to Type of Preoperative Planning eTable 3. Subgroup Analysis for Death in Propensity Score–Matched Patients With Early Surgery eTable 4. Subgroup Analysis for Complications in Propensity Score–Matched Patients With Early Surgery eTable 5. Subgroup Analysis for Death in Propensity Score–Matched Patients Without Early Surgery eTable 6. Subgroup Analysis for Complications in Propensity Score–Matched Patients Without Early Surgery eTable 7. Postoperative Clinical Outcomes in 1-Year Follow-Up of Unmatched Patients eTable 8. Postoperative Clinical Outcomes in 1-Year Follow-Up of All Unmatched Patients eTable 9. Baseline Characteristics of 277 Consecutive Patients Treated by Junior Resident With Use of Computer-Assisted Preoperative Planning eTable 10. Baseline Characteristics of 281 Consecutive Patients Treated by Junior Resident With Use of Conventional Preoperative Planning eFigure 1. Risk of All-Cause 90-Day Mortality in Propensity Score–Matched Patients eFigure 2. Cumulative Incidence of All-Cause 90-Day Complications in Propensity Score–Matched Patients eFigure 3. Learning Curves of Closed Reduction of Fracture Time With Use of Computer-Assisted and Conventional Preoperative Planning eFigure 4. Learning Curves of Number of Fluoroscopies With Use of Computer-Assisted and Conventional Preoperative Planning eFigure 5. Learning Curves of Estimated Blood Loss With Use of Computer-Assisted and Conventional Preoperative Planning [file jamanetwopen-3-e205830-s001.pdf]

Supplementary Online Content

Jia X, Zhang K, Qiang M, Wu Y, Chen Y. Association of computer-assisted virtual preoperative planning with postoperative mortality and complications in older patients with intertrochanteric hip fracture. *JAMA Netw Open*. 2020;3(8):e205830. doi:10.1001/jamanetworkopen.2020.5830

eTable 1. Baseline Demographic Characteristics in Patients With Early Surgery Before and After Propensity Score Matching According to Type of Preoperative Planning

eTable 2. Baseline Demographic Characteristics in Patients Without Early Surgery Before and After Propensity Score Matching According to Type of Preoperative Planning

eTable 3. Subgroup Analysis for Death in Propensity Score–Matched Patients With Early Surgery

eTable 4. Subgroup Analysis for Complications in Propensity Score–Matched Patients With Early Surgery

eTable 5. Subgroup Analysis for Death in Propensity Score–Matched Patients Without Early Surgery

eTable 6. Subgroup Analysis for Complications in Propensity Score–Matched Patients Without Early Surgery

eTable 7. Postoperative Clinical Outcomes in 1-Year Follow-Up of Unmatched Patients

eTable 8. Postoperative Clinical Outcomes in 1-Year Follow-Up of All Unmatched Patients

eTable 9. Baseline Characteristics of 277 Consecutive Patients Treated by Junior Resident With Use of Computer-Assisted Preoperative Planning

eTable 10. Baseline Characteristics of 281 Consecutive Patients Treated by Junior Resident With Use of Conventional Preoperative Planning

eFigure 1. Risk of All-Cause 90-Day Mortality In Propensity Score–Matched Patients

eFigure 2. Cumulative Incidence of All-Cause 90-Day Complications In Propensity Score–Matched Patients

eFigure 3. Learning Curves of Closed Reduction of Fracture Time With Use of Computer-Assisted and Conventional Preoperative Planning

eFigure 4. Learning Curves of Number of Fluoroscopies With Use of Computer-Assisted and Conventional Preoperative Planning

eFigure 5. Learning Curves of Estimated Blood Loss With Use of Computer-Assisted and Conventional Preoperative Planning

This supplementary material has been provided by the authors to give readers additional information about their work.

© 2020 Jia X et al. *JAMA Network Open*.

eTable 1. Baseline Demographic Characteristics in Patients With Early Surgery Before and After Propensity Score Matching According to Type of Preoperative Planning[a]

	Before Propensity Score Matching			After Propensity Score Matching		
Demographics	Computer-Assisted (n = 251)	Conventional (n = 438)	Standardized Difference, %	Computer-assisted (n = 220)	Conventional (n = 220)	Standardized Difference, %
Age, y						
65-69	34 (13.5)	50 (11.4)	6.4	28 (12.7)	27 (12.3)	1.2
70-74	92 (36.7)	173 (39.5)	5.8	77 (35.0)	86 (39.1)	8.5
75-79	77 (30.7)	134 (30.6)	0.2	68 (30.9)	59 (26.8)	9.1
≥80	48 (19.1)	81 (18.5)	1.5	47 (21.4)	48 (21.8)	1.0
Women	164 (65.3)	353 (80.6)	35.0	159 (72.3)	150 (68.2)	9.0
BMI, kg/m^2						
≤18.4	14 (5.6)	34 (7.8)	8.8	13 (5.9)	18 (8.2)	9.0
18.5-23.9	176 (70.1)	252 (57.5)	26.4	150 (68.2)	140 (63.6)	9.7
24.0-27.9	33 (13.1)	90 (20.5)	19.9	31 (14.1)	36 (16.4)	6.4
≥28	28 (11.1)	62 (14.2)	9.3	26 (11.8)	26 (11.8)	0.0
Education						
Primary school	164 (65.3)	218 (49.8)	31.8	142 (64.5)	144 (65.5)	2.1
Junior high school	30 (12.0)	157 (35.8)	58.1	24 (10.9)	26 (11.8)	2.8
Senior high school or above	57 (22.7)	63 (14.4)	21.5	54 (24.6)	50 (22.7)	4.5
Functional status prior to injury						
Independent	186 (74.1)	337 (76.9)	6.5	162 (73.7)	169 (76.8)	7.2
Partially dependent	59 (23.5)	91 (20.8)	6.5	52 (23.6)	44 (20.0)	8.8
Totally dependent	6 (2.3)	10 (2.3)	0.7	6 (2.7)	7 (3.2)	3.0
Injury mechanism						
Falling from height	189 (75.3)	343 (78.3)	7.1	163 (74.1)	171 (77.7)	8.4
Traffic accident	49 (19.5)	46 (10.5)	25.4	47 (21.4)	42 (19.1)	5.7
Other	13 (5.2)	49 (11.2)	22.0	10 (4.5)	7 (3.2)	6.8
Affected side						
Left	150 (59.8)	269 (61.4)	3.3	132 (60.0)	130 (59.1)	1.8
Right	101 (40.2)	169 (38.6)	3.3	88 (40.0)	90 (40.9)	1.8
ASA classification[b]						
Ⅰ-Ⅱ	153 (61.0)	256 (58.4)	5.3	131 (59.6)	137 (62.3)	5.5
Ⅲ	82 (32.7)	144 (32.9)	0.4	74 (33.6)	65 (29.5)	8.8
Ⅳ	16 (6.4)	38 (8.7)	8.7	15 (6.8)	18 (8.2)	5.3
AO/OTA classification[c]						
A1	143 (57.0)	272 (62.1)	10.4	127 (57.7)	125 (56.8)	1.8
A2	92 (36.7)	148 (33.8)	6.07	80 (36.4)	84 (38.2)	3.7
A3	16 (6.4)	18 (4.1)	10.2	13 (5.9)	11 (5.0)	4.0

© 2020 Jia X et al. *JAMA Network Open*.

Medical history						
Alcoholism	34 (13.5)	45 (10.3)	9.9	25 (11.4)	30 (13.6)	6.7
Anemia	20 (8.0)	70 (16.0)	24.8	20 (9.1)	25 (11.4)	7.6
Atrial fibrillation	11 (4.4)	34 (7.8)	14.2	11 (5.0)	14 (6.4)	6.0
Cancer	10 (4.0)	26 (5.9)	8.8	9 (4.1)	12 (5.5)	6.3
Cancer with metastasis	4 (1.6)	10 (2.3)	5.1	3 (1.4)	5 (2.3)	6.5
Chronic kidney disease	16 (6.4)	20 (4.6)	7.9	11 (5.0)	12 (5.5)	2.2
COPD	22 (8.8)	51 (11.6)	9.3	22 (10.0)	21 (9.5)	1.5
Dementia	3 (1.2)	8 (1.8)	4.9	2 (0.9)	2 (0.9)	0.0
Depression	25 (10.0)	21 (4.8)	20.0	17 (7.7)	18 (8.2)	1.8
Diabetes	41 (16.3)	98 (22.4)	15.5	40 (18.2)	45 (20.5)	5.8
Heart failure	13 (5.2)	33 (7.5)	9.4	11 (5.0)	11 (5.0)	0.0
Hypertension	185 (73.7)	311 (71.0)	6.0	161 (73.2)	157 (71.4)	4.0
Intracranial bleeding	3 (1.2)	5 (1.1)	0.9	3 (1.4)	3 (1.4)	0.0
Liver disease	25 (10.0)	55 (12.6)	8.2	25 (11.4)	23 (10.5)	2.9
Myocardial infarction	19 (7.6)	57 (13.0)	17.8	18 (8.2)	20 (10.7)	8.6
Smoking	64 (25.5)	86 (19.6)	7.1	51 (23.2)	49 (22.2)	2.4
Year of surgery						
2009	27 (10.8)	32 (7.3)	12.2	25 (11.4)	23 (10.5)	2.9
2010	24 (9.6)	42 (9.6)	0	20 (9.1)	24 (10.9)	6.0
2011	32 (12.7)	44 (10.0)	8.5	25 (11.4)	29 (13.2)	5.5
2012	23 (9.2)	48 (11.0)	6.0	19 (8.6)	22 (10.0)	4.8
2013	27 (10.8)	47 (10.7)	0.3	26 (11.8)	22 (10.0)	5.8
2014	34 (13.5)	53 (12.1)	4.2	27 (12.3)	25 (11.4)	2.8
2015	32 (12.7)	62 (14.2)	4.4	29 (13.2)	25 (11.4)	5.5
2016	22 (8.8)	47 (10.7)	6.4	20 (9.1)	22 (10.0)	3.1
2017	25 (10.0)	44 (10.0)	0.0	24 (10.9)	21 (9.5)	4.6
2018	5 (2.0)	19 (4.3)	13.2	5 (2.3)	7 (3.2)	5.5

Abbreviation: BMI, body mass index (calculated as weight in kilograms divided by height in maters squared); ASA, American Society of Anesthesiologists; AO/OTA, the AO Foundation/Orthopaedic Trauma Association; COPD, chronic obstructive pulmonary disease.

[a] Data are presented as number (percentage) of patients unless otherwise indicated.

[b] Range, Ⅰ to Ⅳ; higher level indicates higher risk of anesthesia.

[c] Range, A1 to A3; different classification indicates different type of fracture. A1, simple fracture; A2, comminuted fracture involving lateral cortex; A3, reverse oblique fracture.

© 2020 Jia X et al. *JAMA Network Open*.

eTable 2. Baseline Demographic Characteristics in Patients Without Early Surgery Before and After Propensity Score Matching According to Type of Preoperative Planning[a]

	Before Propensity Score Matching			After Propensity Score Matching		
Demographics	Computer-Assisted (n = 214)	Conventional (n = 318)	Standardized Difference, %	Computer-assisted (n = 187)	Conventional (n = 187)	Standardized Difference, %
Age, y						
65-69	30 (14.0)	37 (11.6)	7.2	25 (13.4)	20 (10.7)	8.3
70-74	83 (38.8)	149 (46.9)	16.4	81 (43.3)	90 (48.1)	9.6
75-79	74(34.6)	86 (27.0)	16.5	62 (33.1)	59 (31.6)	3.2
≥80	27 (12.6)	46 (14.5)	5.6	19 (10.2)	18 (9.6)	2.0
Women	151 (70.6)	259 (81.4)	25.5	142 (75.9)	144 (77.0)	2.6
BMI, kg/m^2						
≤18.4	14 (6.5)	33 (10.4)	14.1	12 (6.4)	14 (7.5)	4.3
18.5-23.9	140 (65.4)	181 (56.9)	17.5	121 (64.7)	112 (59.9)	9.9
24.0-27.9	37 (17.3)	61 (19.2)	4.9	32 (17.1)	35 (18.7)	4.2
≥28	23 (10.7)	43 (13.5)	8.6	22 (11.8)	26 (13.9)	6.3
Education						
Primary school	131 (61.2)	163 (51.3)	20.1	111 (59.4)	120 (64.2)	9.9
Junior high school	38 (17.8)	108 (34.0)	37.6	33 (17.6)	30 (16.0)	4.3
Senior high school or above	45 (21.0)	47 (14.8)	16.2	43 (23.0)	37 (19.8)	7.8
Functional status prior to injury						
Independent	148 (69.2)	252 (79.2)	23.0	136 (72.7)	140 (74.9)	5.0
Partially dependent	64 (29.9)	63 (19.8)	23.5	50 (26.7)	45 (24.1)	6.0
Totally dependent	2 (0.9)	3 (0.9)	0.0	1 (0.6)	2 (1.0)	4.5
Injury mechanism						
Falling from height	142 (66.4)	232 (73.0)	14.4	130 (69.5)	137 (73.3)	8.4
Traffic accident	43 (20.1)	57 (17.9)	5.6	34 (18.2)	32 (17.1)	2.9
Other	29 (13.6)	29 (9.1)	14.2	23 (12.3)	18 (9.6)	8.6
Affected side						
Left	115 (53.7)	222 (69.8)	33.6	110 (58.8)	108 (57.8)	2.0
Right	99 (46.3)	96 (30.2)	33.6	77 (41.2)	79 (42.2)	2.0
ASA classification[b]						
Ⅰ-Ⅱ	116 (54.2)	190 (59.7)	11.1	105 (56.2)	110 (58.8)	5.3
Ⅲ	86 (40.2)	106 (33.3)	14.3	72 (38.5)	64 (34.2)	8.9
Ⅳ	12 (5.6)	22 (6.9)	5.4	10 (5.3)	13 (7.0)	7.1
AO/OTA classification[c]						
A1	134 (62.6)	204 (64.2)	3.3	116 (62.0)	122 (65.2)	6.7
A2	66 (30.8)	95 (29.9)	2.0	59 (31.6)	51 (27.3)	9.4
A3	14 (6.5)	19 (6.0)	2.1	12 (6.4)	14 (7.5)	4.3

© 2020 Jia X et al. *JAMA Network Open*.

Medical history						
Alcoholism	36 (16.8)	31 (9.7)	21.1	25 (13.4)	24 (12.8)	1.8
Anemia	25 (11.7)	43 (13.5)	5.4	21 (11.2)	23 (12.3)	3.4
Atrial fibrillation	7 (3.3)	20 (6.3)	14.1	6 (3.2)	8 (4.3)	5.8
Cancer	13 (6.1)	19 (6.0)	0.4	10 (5.3)	11 (5.9)	2.6
Cancer with metastasis	6 (2.8)	5 (1.6)	8.2	4 (2.1)	4 (2.1)	0.0
Chronic kidney disease	17 (7.9)	18 (5.7)	8.7	13 (7.0)	11 (5.9)	4.3
COPD	18 (8.4)	34 (10.7)	7.8	18 (9.6)	16 (8.6)	3.5
Dementia	2 (0.9)	5 (1.6)	6.3	2 (1.1)	1 (0.5)	6.3
Depression	12 (5.6)	9 (2.8)	14.0	8 (4.3)	9 (4.8)	2.4
Diabetes	42 (19.6)	68 (21.4)	4.5	35 (18.7)	36 (19.3)	1.5
Heart failure	10 (4.7)	28 (8.8)	16.4	10 (5.3)	13 (7.0)	6.9
Hypertension	163 (76.2)	216 (67.9)	18.6	141 (75.4)	140 (74.9)	1.2
Intracranial bleeding	1 (0.5)	7 (2.2)	14.8	1 (0.5)	0 (0.0)	10.4
Liver disease	23 (10.7)	44 (13.8)	9.5	21 (11.2)	21 (11.2)	0.0
Myocardial infarction	15 (7.0)	33 (10.4)	12.1	14 (6.4)	12 (5.5)	3.8
Smoking	62 (29.0)	72 (22.6)	14.7	49 (26.2)	47 (25.1)	2.5
Year of surgery						
2009	16 (7.5)	32 (10.1)	9.2	15 (8.0)	14 (7.5)	1.9
2010	27 (12.6)	33 (10.4)	6.9	20 (10.7)	24 (12.8)	6.5
2011	23 (10.7)	32 (10.1)	2.0	20 (10.7)	23 (12.3)	5.0
2012	31 (14.5)	37 (11.6)	8.6	28 (15.0)	27 (14.4)	1.7
2013	23 (10.7)	40 (12.6)	5.9	21 (11.2)	25 (13.4)	6.7
2014	21 (9.8)	32 (10.1)	1.0	20 (10.7)	20 (10.7)	0.0
2015	29 (13.6)	24 (7.5)	20.0	25 (13.4)	20 (10.7)	8.3
2016	19 (8.9)	37 (11.6)	8.9	14 (7.5)	12 (6.4)	4.3
2017	18 (8.4)	41 (12.9)	14.6	17 (9.1)	15 (8.0)	3.9
2018	7 (3.3)	10 (3.1)	1.1	7 (3.7)	7 (3.7)	0.0

Abbreviation: BMI, body mass index (calculated as weight in kilograms divided by height in maters squared); ASA, American Society of Anesthesiologists; AO/OTA, the AO Foundation/Orthopaedic Trauma Association; COPD, chronic obstructive pulmonary disease.

[a] Data are presented as number (percentage) of patients unless otherwise indicated.

[b] Range, Ⅰ to Ⅳ; higher level indicates higher risk of anesthesia.

[c] Range, A1 to A3; different classification indicates different type of fracture. A1, simple fracture; A2, comminuted fracture involving lateral cortex; A3, reverse oblique fracture.

© 2020 Jia X et al. *JAMA Network Open*.

eTable 3. Subgroup Analysis for Death in Propensity Score–Matched Patients With Early Surgery

	Computer-Assisted			Conventional				
Demographics	No. of Patients	No. of Events	Event Rate	No. of Patients	No. of Events	Event Rate	HR (95% CI)	*P* Value
All patients	220	16	7.3	220	29	13.2	0.52 (0.27-0.98)	.04
Age, y								
65-69	28	0	0.0	27	3	11.1	0.56 (0.45-1.03)	.07
70-74	77	2	2.6	86	5	5.8	0.83 (0.66-1.39)	.31
75-79	68	6	8.8	59	10	16.9	0.70 (0.56-1.18)	.17
≥80	47	8	17.0	48	11	22.9	0.72 (0.58-1.22)	.47
Sex								
Male	61	4	6.6	70	10	14.3	0.79 (0.63-1.33)	.15
Female	159	12	7.5	150	19	12.7	0.59 (0.47-1.15)	.13
Functional status prior to injury								
Independent	162	10	6.2	169	16	9.5	0.53 (0.42-1.28)	.27
Partially dependent	52	5	9.6	44	10	22.7	0.55 (0.44-1.09)	.08
Totally dependent	6	1	16.7	7	3	42.9	0.75 (0.60-1.26)	.31
ASA classification[a]								
Ⅰ-Ⅱ	131	11	8.4	137	18	13.1	0.83 (0.66-1.39)	.21
Ⅲ	74	5	6.8	65	8	12.3	0.65 (0.52-1.08)	.26
Ⅳ	15	0	0.0	18	3	16.7	0.60 (0.48-1.01)	.10
AO/OTA classification[b]								
A1	127	8	6.3	125	12	9.6	0.79 (0.63-1.33)	.33
A2	80	6	7.5	84	12	14.3	0.73 (0.59-1.23)	.17
A3	13	2	15.4	11	5	45.5	0.76 (0.61-1.28)	.11
Anemia								
No	200	6	3.0	195	11	5.6	0.97 (0.77-1.21)	.20
Yes	20	10	50.0	25	18	72.0	0.92 (0.73-1.44)	.13
Atrial fibrillation								
No	209	7	3.3	206	15	7.3	0.81 (0.65-1.36)	.07
Yes	11	9	81.8	14	14	100.0	0.41 (0.33-1.17)	.10
Chronic kidney disease								
No	209	10	4.8	208	19	9.1	0.71 (0.57-1.20)	.08
Yes	11	6	54.5	12	10	83.3	0.76 (0.61-1.27)	.13
Heart failure								
No	209	11	5.3	209	21	10.0	0.50 (0.40-1.04)	.07
Yes	11	5	45.5	11	8	72.7	0.80 (0.64-1.34)	.19
Intracranial bleeding								
No	217	16	7.4	217	27	12.4	0.56 (0.29-1.07)	.08
Yes	3	0	0.0	3	2	66.7	0.71 (0.64-1.12)	.39
Myocardial infarction								
No	202	11	5.4	200	18	9.0	0.86 (0.69-1.24)	.17

© 2020 Jia X et al. *JAMA Network Open*.

Yes		18		5		27.8		20		11		55.0	0.79 (0.64-1.33)	.09

ASA indicates American Society of Anesthesiologists; AO/OTA, the AO Foundation/Orthopaedic Trauma Association; HR, hazard ratio; CI, confidence interval.
[a] Range, Ⅰ to Ⅳ; higher level indicates higher risk of anesthesia.
[b] Range, A1 to A3; different classification indicates different type of fracture. A1, simple fracture; A2, comminuted fracture involving lateral cortex; A3, reverse oblique fracture.

© 2020 Jia X et al. *JAMA Network Open*.

eTable 4. Subgroup Analysis for Complications in Propensity Score–Matched Patients With Early Surgery

	Computer-Assisted			Conventional				
Demographics	No. of Patients	No. of Events	Event Rate	No. of Patients	No. of Events	Event Rate	HR (95% CI)	*P* Value
All patients	220	12	5.5	220	28	12.7	0.40 (0.20-0.80)	.01
Age, y								
65-69	28	1	3.6	27	4	14.8	0.51 (0.43-1.03)	.15
70-74	77	2	2.6	86	6	7.0	0.66 (0.55-1.31)	.20
75-79	68	4	5.9	59	8	13.6	0.70 (0.59-1.40)	.14
≥80	47	5	10.6	48	10	20.8	0.75 (0.63-1.50)	.17
Sex								
Male	61	3	4.9	70	11	15.7	0.58 (0.48-1.15)	.09
Female	159	9	5.7	150	17	11.3	0.77 (0.65-1.54)	.07
Functional status prior to injury								
Independent	162	6	3.7	169	14	8.3	0.73 (0.61-1.45)	.08
Partially dependent	52	3	5.8	44	8	18.2	0.58 (0.48-1.15)	.06
Totally dependent	6	3	50.0	7	6	85.7	0.47 (0.39-1.03)	.16
ASA classification[a]								
Ⅰ-Ⅱ	131	5	3.8	137	11	8.0	0.76 (0.63-1.51)	.15
Ⅲ	74	3	4.1	65	8	12.3	0.60 (0.50-1.20)	.07
Ⅳ	15	4	26.7	18	9	50.0	0.66 (0.56-1.33)	.17
AO/OTA classification[b]								
A1	127	6	4.7	125	13	10.4	0.73 (0.61-1.45)	.09
A2	80	3	3.8	84	10	11.9	0.59 (0.49-1.18)	.05
A3	13	3	23.1	11	5	45.5	0.66 (0.55-1.32)	.25
Anemia								
No	200	6	3.0	195	14	7.2	0.70 (0.59-1.40)	.06
Yes	20	6	30.0	25	14	56.0	0.64 (0.54-1.27)	.08
Atrial fibrillation								
No	209	7	3.3	206	16	7.8	0.71 (0.60-1.42)	.05
Yes	11	5	45.5	14	12	85.7	0.44 (0.37-1.27)	.08
Chronic kidney disease								
No	209	10	4.8	208	20	9.6	0.77 (0.65-1.54)	.06
Yes	11	2	18.2	12	8	66.7	0.41 (0.35-1.15)	.06
Heart failure								
No	209	9	4.3	209	19	9.1	0.75 (0.63-1.50)	.051
Yes	11	3	27.3	11	9	81.8	0.38 (0.32-0.77)	.03
Intracranial bleeding								
No	217	11	5.1	217	25	11.5	0.41 (0.20-0.86)	.02
Yes	3	1	33.3	3	3	100.0	0.33 (0.11-1.45)	.40
Myocardial infarction								
No	202	4	2.0	200	12	6.0	0.65 (0.54-1.29)	.07

© 2020 Jia X et al. *JAMA Network Open*.

Yes		18		8		44.4		20		16		80.0	0.50 (0.42-1.18)	.05

ASA indicates American Society of Anesthesiologists; AO/OTA, the AO Foundation/Orthopaedic Trauma Association; HR, hazard ratio; CI, confidence interval.

[a] Range, Ⅰ to Ⅳ; higher level indicates higher risk of anesthesia.

[b] Range, A1 to A3; different classification indicates different type of fracture. A1, simple fracture; A2, comminuted fracture involving lateral cortex; A3, reverse oblique fracture.

© 2020 Jia X et al. *JAMA Network Open*.

eTable 5. Subgroup Analysis for Death in Propensity Score–Matched Patients Without Early Surgery

	Computer-Assisted			Conventional				
Demographics	No. of Patients	No. of Events	Event Rate	No. of Patients	No. of Events	Event Rate	HR (95% CI)	*P* Value
All patients	187	21	11.2	187	26	13.9	0.78 (0.67-1.16)	.44
Age, y								
65-69	25	3	12.0	20	4	20.0	0.55 (0.47-1.20)	.46
70-74	81	6	7.4	90	5	5.6	1.36 (1.17-1.74)	.62
75-79	62	3	4.8	59	4	6.8	0.70 (0.60-1.24)	.65
≥80	19	9	47.4	18	13	72.2	0.85 (0.73-1.26)	.12
Sex								
Male	45	10	22.2	43	14	32.6	0.61 (0.53-1.13)	.28
Female	142	11	7.7	144	12	8.3	0.92 (0.79-1.37)	.86
Functional status prior to injury								
Independent	136	10	7.4	140	12	8.6	0.85 (0.73-1.42)	.71
Partially dependent	50	10	20.0	45	13	28.9	0.62 (0.53-1.22)	.31
Totally dependent	1	1	100.0	2	1	50.0	1.14 (0.98-1.68)	.39
ASA classification[a]								
Ⅰ-Ⅱ	105	6	5.7	110	10	9.1	0.61 (0.52-1.19)	.35
Ⅲ	72	7	9.7	64	9	14.1	0.66 (0.57-1.23)	.43
Ⅳ	10	8	80.0	13	7	53.8	1.24 (0.78-1.59)	.19
AO/OTA classification[b]								
A1	116	8	6.9	122	10	8.2	0.83 (0.71-1.23)	.71
A2	59	8	13.6	51	9	17.6	0.73 (0.63-1.08)	.55
A3	12	5	41.7	14	7	50.0	0.71 (0.61-1.06)	.67
Anemia								
No	166	11	6.6	164	14	8.5	0.76 (0.65-1.12)	.51
Yes	21	10	47.6	23	12	52.2	0.83 (0.72-1.23)	.76
Atrial fibrillation								
No	181	16	8.8	179	19	10.6	0.82 (0.70-1.21)	.57
Yes	6	5	83.3	8	7	87.5	0.71 (0.61-1.06)	.83
Chronic kidney disease								
No	174	11	6.3	176	17	9.7	0.63 (0.54-1.25)	.25
Yes	13	10	76.9	11	9	81.8	0.74 (0.64-1.17)	.77
Heart failure								
No	177	13	7.3	174	16	9.2	0.78 (0.67-1.16)	.53
Yes	10	8	80.0	13	10	76.9	1.20 (1.03-1.78)	.86
Intracranial bleeding								
No	186	20	10.8	187	26	13.9	0.75 (0.64-1.10)	.36
Yes	1	1	100.0	0	0	0.0	NA	NA
Myocardial infarction								
No	173	17	9.8	175	18	10.3	0.95 (0.82-1.41)	.89

© 2020 Jia X et al. *JAMA Network Open*.

Yes		14		4		28.6		12		8		66.7	0.69 (0.59-1.02)	.11

ASA indicates American Society of Anesthesiologists; AO/OTA, the AO Foundation/Orthopaedic Trauma Association; HR, hazard ratio; CI, confidence interval; NA, not available.

[a] Range, Ⅰ to Ⅳ; higher level indicates higher risk of anesthesia.

[b] Range, A1 to A3; different classification indicates different type of fracture. A1, simple fracture; A2, comminuted fracture involving lateral cortex; A3, reverse oblique fracture.

© 2020 Jia X et al. *JAMA Network Open*.

eTable 6. Subgroup Analysis for Complications in Propensity Score–Matched Patients Without Early Surgery

	Computer-Assisted			Conventional				
Demographics	No. of Patients	No. of Events	Event Rate	No. of Patients	No. of Events	Event Rate	HR (95% CI)	*P* Value
All patients	187	13	7.0	187	16	8.6	0.80 (0.69-1.18)	.56
Age, y								
65-69	25	4	16.0	20	4	20.0	0.76 (0.66-1.13)	.73
70-74	81	4	4.9	90	5	5.6	0.88 (0.76-1.31)	.86
75-79	62	3	4.8	59	6	10.2	0.62 (0.53-1.10)	.26
≥80	19	2	10.5	18	1	5.6	0.87 (0.75-1.29)	.58
Sex								
Male	45	4	8.9	43	6	14.0	0.60 (0.52-1.11)	.45
Female	142	9	6.3	144	10	6.9	0.91 (0.78-1.34)	.84
Functional status prior to injury								
Independent	136	6	4.4	140	9	6.4	0.67 (0.58-1.13)	.46
Partially dependent	50	6	12.0	45	6	13.3	0.89 (0.76-1.31)	.85
Totally dependent	1	1	100.0	2	1	50.0	1.24 (1.07-1.84)	.39
ASA classification[a]								
Ⅰ-Ⅱ	105	6	5.7	110	5	4.5	1.27 (1.09-1.88)	.70
Ⅲ	72	5	6.9	64	5	7.8	0.88 (0.76-1.30)	.85
Ⅳ	10	2	20.0	13	6	46.2	0.72 (0.61-1.06)	.19
AO/OTA classification[b]								
A1	116	6	5.2	122	7	5.7	0.90 (0.77-1.33)	.85
A2	59	4	6.8	51	5	9.8	0.77 (0.66-1.14)	.56
A3	12	3	25.0	14	4	28.6	0.83 (0.72-1.23)	.84
Anemia								
No	166	9	5.4	164	10	6.1	0.88 (0.76-1.31)	.79
Yes	21	4	19.0	23	6	26.1	0.77 (0.66-1.14)	.58
Atrial fibrillation								
No	181	10	5.5	179	11	6.1	0.89 (0.77-1.32)	.80
Yes	6	3	50.0	8	5	62.5	0.71 (0.61-1.04)	.64
Chronic kidney disease								
No	174	9	5.2	176	9	5.1	1.01 (0.87-1.50)	.98
Yes	13	4	30.8	11	7	63.6	0.75 (0.65-1.12)	.11
Heart failure								
No	177	9	5.1	174	10	5.7	0.88 (0.76-1.30)	.78
Yes	10	4	40.0	13	6	46.2	0.78 (0.67-1.15)	.77
Intracranial bleeding								
No	186	12	6.5	187	16	8.6	0.74 (0.63-1.09)	.44
Yes	1	1	100.0	0	0	0.0	NA	NA
Myocardial infarction								
No	173	7	4.0	175	9	5.1	0.78 (0.67-1.15)	.63

© 2020 Jia X et al. *JAMA Network Open*.

Yes		14		6		42.9		12		7		58.3	0.84 (0.72-1.24)	.43

ASA indicates American Society of Anesthesiologists; AO/OTA, the AO Foundation/Orthopaedic Trauma Association; HR, hazard ratio; CI, confidence interval; NA, not available.

[a] Range, Ⅰ to Ⅳ; higher level indicates higher risk of anesthesia.

[b] Range, A1 to A3; different classification indicates different type of fracture. A1, simple fracture; A2, comminuted fracture involving lateral cortex; A3, reverse oblique fracture.

© 2020 Jia X et al. *JAMA Network Open*.

eTable 7. Postoperative Clinical Outcomes in 1-Year Follow-up of Unmatched Patients

Outcome		Computer-Assisted[a]		Conventional[a]		*P* Value
Harris[b]						
Overall (n1=390; n2=636)		66.8 ± 9.6		66.1 ± 6.8		.16
≤24 hours surgery (n1=217; n2=375)		69.7 ± 7.4		68.9 ± 6.6		.06
>24 hours surgery (n1=173; n2=261)		64.1 ± 9.7		63.5 ± 7.4		.25
SF-36 PCS[c]						
Overall (n1=390; n2=636)		70.3 ± 6.5		69.9 ± 5.8		.29
≤24 hours surgery (n1=217; n2=375)		73.5 ± 5.4		72.8 ± 6.8		.08
>24 hours surgery (n1=173; n2=261)		67.4 ± 6.8		66.9 ± 6.3		.22
VAS Score[d]						
Overall (n1=390; n2=636)		3.5 ± 1.3		3.6 ± 1.2		.20
≤24 hours surgery (n1=217; n2=375)		3.1 ± 1.1		3.2 ± 1.4		.22
>24 hours surgery (n1=173; n2=261)		3.8 ± 1.6		3.9 ± 1.2		.24

Abbreviation: SF-36 PCS, short-form-36 physical component summary; VAS, visual analog scale.
[a] Data are shown as mean ± standard deviation.
[b] Range, 0 to 100; higher score indicates the better function of hip.
[c] Range, 0 to 100; higher score indicates the better of physical function.
[d] Range, 0 to 10; higher score indicates the more intense pain.
n1, the number of patients in the computer-assisted group; n2, the number of patients in the conventional group.

© 2020 Jia X et al. *JAMA Network Open*.

eTable 8. Postoperative Clinical Outcomes in 1-Year Follow-up of All Unmatched Patients

Outcome		Computer-Assisted[a] (n=465)	Conventional[a] (n=756)	*P* Value
Harris[b]				
Overall (n1=465; n2=756)		67.0 ± 8.3	66.6 ± 6.8	0.36
≤24 hours (n1=251; n2=438)		69.6 ± 7.0	68.9 ± 6.0	0.17
>24 hours (n1=214; n2=318)		64.0 ± 8.6	63.4 ± 6.6	0.44
SF-36 PCS[c]				
Overall (n1=465; n2=756)		70.5 ± 6.4	70.3 ± 6.7	0.46
≤24 hours (n1=251; n2=438)		73.4 ± 5.1	72.7 ± 6.3	0.14
>24 hours (n1=214; n2=318)		67.2 ± 6.4	66.9 ± 5.8	0.49
VAS Score[d]				
Overall (n1=465; n2=756)		3.4 ± 1.3	3.5 ± 1.3	0.84
≤24 hours (n1=251; n2=438)		3.1 ± 1.1	3.2 ± 1.3	0.73
>24 hours (n1=214; n2=318)		3.8 ± 1.4	3.9 ± 1.1	0.63

Abbreviation: SF-36 PCS, short-form-36 physical component summary; VAS, visual analog scale.
a Data are shown as mean ± standard deviation.
b Range, 0 to 100; higher score indicates the better function of hip.
c Range, 0 to 100; higher score indicates the better of physical function.
d Range, 0 to 10; higher score indicates the more intense pain.
n1, the number of patients in the computer-assisted group; n2, the number of patients in the conventional group.

© 2020 Jia X et al. *JAMA Network Open*.

eTable 9. Baseline Characteristics of 277 Consecutive Patients Treated by Junior Resident With Use of Computer-Assisted Preoperative Planning[a]

		Group (Case)											
	Total	1 (1-25)	2 (26-50)	3 (51-75)	4 (76-100)	5 (101-125)	6 (126-150)	7 (151-175)	8 (176-200)	9 (201-225)	10 (226-250)	11 (251-277)	*P* Value
Sex													0.986
Male	82 (29.6)	7 (28.0)	6 (24.0)	8 (32.0)	9 (36.0)	8 (32.0)	8 (32.0)	9 (36.0)	7 (28.0)	6 (24.0)	8 (32.0)	6 (22.2)	
Female	195 (70.4)	18 (72.0)	19 (76.0)	17 (68.0)	16 (64.0)	17 (68.0)	17 (68.0)	16 (64.0)	18 (72.0)	19 (76.0)	17 (68.0)	21 (77.8)	
Age (yr)													1.000
65-69	70 (25.3)	7 (28.0)	8 (32.0)	7 (28.0)	5 (20.0)	6 (24.0)	6 (24.0)	9 (36.0)	6 (24.0)	6 (24.0)	6 (24.0)	9 (36.0)	
70-74	98 (35.4)	9 (36.0)	8 (32.0)	7 (28.0)	9 (36.0)	11 (40.7)	9 (36.0)	9 (36.0)	7 (28.0)	8 (32.0)	9 (36.0)	11 (40.7)	
75-79	61 (22.0)	4 (16.0)	5 (20.0)	6 (24.0)	6 (24.0)	4 (16.0)	6 (24.0)	2 (8.0)	6 (24.0)	8 (32.0)	6 (24.0)	3 (11.1)	
≥80	48 (17.3)	5 (20.0)	4 (16.0)	5 (20.0)	5 (20.0)	4 (16.0)	3 (12.0)	5 (20.0)	6 (24.0)	3 (12.0)	4 (16.0)	4 (14.8)	
BMI (kg/m^2)	24.8 ± 1.7	25.1 ± 1.4	25.0 ± 1.8	25.2 ± 1.2	23.8 ± 1.8	25.0 ± 1.9	25.1 ± 1.4	24.8 ± 1.7	24.1 ± 1.2	24.8 ± 1.4	24.6 ± 1.9	24.1 ± 2.2	0.075
Education													0.936
Primary school	153 (55.2)	14 (56.0)	15 (60.0)	16 (64.0)	13 (52.0)	12 (48.0)	13 (52.0)	11 (44.0)	15 (60.0)	14 (56.0)	14 (56.0)	16 (59.3)	
Junior high school	85 (30.7)	8 (32.0)	8 (32.0)	7 (28.0)	7 (28.0)	8 (32.0)	6 (24.0)	8 (32.0)	9 (36.0)	8 (32.0)	7 (28.0)	9 (33.3)	
Senior high school or above	39 (14.1)	3 (12.0)	2 (8.0)	2 (8.0)	5 (20.0)	5 (20.0)	6 (24.0)	6 (24.0)	1 (4.0)	3 (12.0)	4 (16.0)	2 (7.4)	
Diabetes	84 (30.3)	6 (24.0)	7 (28.0)	8 (32.0)	7 (28.0)	6 (24.0)	7 (28.0)	9 (36.0)	7 (28.0)	9 (36.0)	8 (32.0)	10 (37.0)	0.989
Smoking status	14 (5.1)	1 (4.0)	2 (8.0)	0 (0.0)	3 (12.0)	0 (0.0)	1 (4.0)	2 (8.0)	1 (4.0)	1 (4.0)	1 (4.0)	2 (7.4)	0.755
Steroid use	16 (5.8)	2 (8.0)	2 (8.0)	1 (4.0)	0 (0.0)	1 (4.0)	2 (8.0)	1 (4.0)	2 (8.0)	1 (4.0)	2 (8.0)	2 (7.4)	0.971
Functional status prior to injury													0.799
Independent	203 (73.3)	17 (68.0)	18 (72.0)	16 (64.0)	17 (68.0)	17 (68.0)	22 (88.0)	20 (80.0)	18 (72.0)	20 (80.0)	15 (60.0)	23 (85.2)	
Partially dependent	66 (23.8)	6 (24.0)	7 (28.0)	8 (32.0)	7 (28.0)	7 (28.0)	3 (12.0)	4 (16.0)	6 (24.0)	5 (20.0)	9 (36.0)	4 (14.8)	
Totally dependent	8 (2.9)	2 (8.0)	0 (0.0)	1 (4.0)	1 (4.0)	1 (4.0)	0 (0.0)	1 (4.0)	1 (4.0)	0 (0.0)	1 (4.0)	0 (0.0)	
Injury mechanism													0.736
Falling from height	221 (79.8)	20 (80.0)	22 (88.0)	18 (72.0)	19 (76.0)	20 (80.0)	21 (84.0)	17 (68.0)	19 (76.0)	20 (80.0)	22 (88.0)	23 (85.2)	

© 2020 Jia X et al. *JAMA Network Open*.

Traffic accident	43 (15.5)	4 (16.0)	1 (4.0)	5 (20.0)	6 (24.0)	4 (16.0)	3 (12.0)	7 (28.0)	6 (24.0)	3 (12.0)	2 (8.0)	2 (7.4)	
Other	13 (4.7)	1 (4.0)	2 (8.0)	2 (8.0)	0 (0.0)	1 (4.0)	1 (4.0)	1 (4.0)	0 (0.0)	2 (8.0)	1 (4.0)	2 (7.4)	
Affected side													0.854
Left	157 (56.7)	13 (52.0)	12 (48.0)	15 (60.0)	16 (64.0)	13 (52.0)	15 (60.0)	16 (64.0)	17 (68.0)	15 (60.0)	12 (48.0)	13 (48.1)	
Right	120 (43.3)	12 (48.0)	13 (52.0)	10 (40.0)	9 (36.0)	12 (48.0)	10 (40.0)	9 (36.0)	8 (32.0)	10 (40.0)	13 (52.0)	14 (51.9)	
ASA classification[b]													0.932
Ⅰ-Ⅱ	165 (59.6)	15 (60.0)	16 (64.0)	17 (68.0)	14 (56.0)	15 (60.0)	13 (52.0)	12 (48.0)	15 (60.0)	14 (56.0)	16 (64.0)	18 (66.7)	
Ⅲ	102 (36.8)	9 (36.0)	9 (36.0)	6 (24.0)	10 (40.0)	9 (36.0)	12 (48.0)	13 (52.0)	9 (36.0)	10 (40.0)	8 (32.0)	7 (25.9)	
Ⅳ	10 (3.6)	1 (4.0)	0 (0.0)	2 (8.0)	1 (4.0)	1 (4.0)	0 (0.0)	0 (0.0)	1 (4.0)	1 (4.0)	1 (4.0)	2 (7.4)	
AO/OTA classification[c]													0.796
A1	141 (50.9)	14 (56.0)	14 (56.0)	9 (36.0)	13 (52.0)	17 (68.0)	15 (60.0)	11 (44.0)	8 (32.0)	14 (56.0)	15 (60.0)	11 (40.7)	
A2	107 (38.6)	9 (36.0)	8 (32.0)	12 (48.0)	10 (40.0)	7 (28.0)	8 (32.0)	11 (44.0)	13 (52.0)	8 (32.0)	9 (36.0)	12 (44.4)	
A3	29 (10.5)	2 (8.0)	3 (12.0)	4 (16.0)	2 (8.0)	1 (4.0)	2 (8.0)	3 (12.0)	4 (16.0)	3 (12.0)	1 (4.0)	4 (14.8)	
Time to operation (hours)													0.996
≤24	268 (96.8)	24 (96.0)	24 (96.0)	24 (96.0)	24 (96.0)	25 (100.0)	24 (96.0)	24 (96.0)	25 (100.0)	24 (96.0)	24 (96.0)	26 (96.3)	
>24	9 (3.2)	1 (4.0)	1 (4.0)	1 (4.0)	1 (4.0)	0 (0.0)	1 (4.0)	1 (4.0)	0 (0.0)	1 (4.0)	1 (4.0)	1 (3.7)	

Abbreviation: BMI, body mass index (calculated as weight in kilograms divided by height in maters squared); ASA, American Society of Anesthesiologists; AO/OTA, the AO Foundation/Orthopaedic Trauma Association.

[a] Data are shown as number (percentage) if the variable is categorical and as mean ± standard deviation if continuous and normally distributed.

[b] Range, Ⅰ to Ⅳ; higher level indicates higher risk of anesthesia.

[c] Range, A1 to A3; different classification indicates different type of fracture. A1, simple fracture; A2, comminuted fracture involving lateral cortex; A3, reverse oblique fracture.

© 2020 Jia X et al. *JAMA Network Open*.

eTable 10. Baseline Characteristics of 281 Consecutive Patients Treated by Junior Resident With Use of Conventional Preoperative Planning[a]

		Group (Case)												
	Total	1 (1-25)	2 (26-50)	3 (51-75)	4 (76-100)	5 (101-125)	6 (126-150)	7 (151-175)	8 (176-200)	9 (201-225)	10 (226-250)	11 (251-275)	12 (276-281)	*P* Value
Sex														0.998
Male	87 (31.0)	7 (28.0)	8 (32.0)	9 (36.0)	7 (28.0)	9 (36.0)	7 (28.0)	9 (36.0)	8 (32.0)	8 (32.0)	7 (28.0)	7 (28.0)	1 (16.7)	
Female	194 (69.0)	18 (72.0)	17 (68.0)	16 (64.0)	18 (72.0)	16 (64.0)	18 (72.0)	16 (64.0)	17 (68.0)	17 (68.0)	18 (72.0)	18 (72.0)	5 (83.3)	
Age (yr)														0.998
65-69	64 (22.8)	5 (20.0)	5 (20.0)	7 (28.0)	8 (32.0)	8 (32.0)	6 (24.0)	5 (20.0)	6 (24.0)	7 (28.0)	6 (24.0)	4 (16.0)	1 (16.7)	
70-74	100 (35.6)	9 (36.0)	10 (40.0)	11 (44.0)	9 (36.0)	9 (36.0)	8 (32.0)	7 (28.0)	8 (32.0)	8 (32.0)	9 (36.0)	9 (36.0)	2 (33.3)	
75-89	79 (28.1)	8 (32.0)	7 (28.0)	3 (12.0)	4 (16.0)	3 (12.0)	7 (28.0)	11 (44.0)	8 (32.0)	7 (28.0)	7 (28.0)	9 (36.0)	2 (33.3)	
≥80	38 (13.5)	3 (12.0)	3 (12.0)	4 (16.0)	4 (16.0)	5 (20.0)	4 (16.0)	2 (8.0)	3 (12.0)	3 (12.0)	3 (12.0)	3 (12.0)	1 (16.7)	
BMI (kg/m^2)	24.4 ± 1.6	24.1 ± 1.8	24.1 ± 1.9	24.3 ± 1.3	24.3 ± 1.5	24.1 ± 1.3	24.0 ± 2.0	25.2 ± 1.6	24.1 ± 1.0	25.1 ± 1.7	25.0 ± 1.6	24.1 ± 2.0	24.3 ± 1.2	0.089
Education														0.835
Primary school	161 (57.3)	16 (64.0)	15 (60.0)	14 (56.0)	13 (52.0)	12 (48.0)	13 (52.0)	15 (60.0)	16 (64.0)	14 (56.0)	15 (60.0)	14 (56.0)	4 (66.7)	
Junior high school	78 (27.8)	8 (32.0)	7 (28.0)	7 (28.0)	6 (24.0)	6 (24.0)	5 (20.0)	7 (28.0)	8 (32.0)	9 (36.0)	7 (28.0)	7 (28.0)	1 (16.7)	
Senior high school or above	42 (14.9)	1 (4.0)	3 (12.0)	4 (16.0)	6 (24.0)	7 (28.0)	7 (28.0)	3 (12.0)	1 (4.0)	2 (8.0)	3 (12.0)	4 (16.0)	1 (16.7)	
Diabetes	81 (28.8)	6 (24.0)	7 (28.0)	10 (40.0)	9 (36.0)	9 (36.0)	5 (20.0)	6 (24.0)	5 (20.0)	6 (24.0)	7 (28.0)	8 (32.0)	3 (50.0)	0.801
Smoking status	13 (4.6)	1 (4.0)	1 (4.0)	0 (0.0)	0 (0.0)	1 (4.0)	2 (8.0)	1 (4.0)	3 (12.0)	2 (8.0)	1 (4.0)	1 (4.0)	0 (0.0)	0.781
Steroid use	18 (6.4)	1 (4.0)	0 (0.0)	2 (8.0)	3 (12.0)	2 (8.0)	1 (4.0)	2 (8.0)	2 (8.0)	1 (4.0)	1 (4.0)	3 (12.0)	0 (0.0)	0.865
Functional status prior to injury														0.898
Independent	210 (74.7)	15 (60.0)	20 (80.0)	21 (84.0)	18 (72.0)	19 (76.0)	20 (80.0)	21 (84.0)	17 (68.0)	16 (64.0)	18 (72.0)	20 (80.0)	5 (83.3)	
Partially dependent	65 (23.1)	9 (36.0)	5 (20.0)	4 (16.0)	6 (24.0)	6 (24.0)	4 (16.0)	3 (12.0)	7 (28.0)	9 (36.0)	6 (24.0)	5 (20.0)	1 (16.7)	
Totally dependent	6 (2.1)	1 (4.0)	0 (0.0)	0 (0.0)	1 (4.0)	0 (0.0)	1 (4.0)	1 (4.0)	1 (4.0)	0 (0.0)	1 (4.0)	0 (0.0)	0 (0.0)	

© 2020 Jia X et al. *JAMA Network Open*.

Injury mechanism														0.508
Falling from height	232 (82.6)	22 (88.0)	23 (92.0)	20 (80.0)	18 (72.0)	17 (68.0)	19 (76.0)	23 (92.0)	23 (92.0)	24 (96.0)	20 (80.0)	18 (72.0)	5 (83.3)	
Traffic accident	41 (14.6)	2 (8.0)	1 (4.0)	5 (20.0)	5 (20.0)	7 (28.0)	5 (20.0)	2 (8.0)	2 (8.0)	1 (4.0)	1 (4.0)	6 (24.0)	1 (16.7)	
Other	8 (2.8)	1 (4.0)	1 (4.0)	0 (0.0)	2 (8.0)	1 (4.0)	1 (4.0)	0 (0.0)	0 (0.0)	0 (0.0)	0 (0.0)	1 (4.0)	0 (0.0)	
Affected side														0.937
Left	149 (53.0)	12 (48.0)	13 (52.0)	11 (44.0)	12 (48.0)	13 (52.0)	15 (60.0)	17 (68.0)	15 (60.0)	13 (52.0)	12 (48.0)	13 (52.0)	3 (50.0)	
Right	132 (47.0)	13 (52.0)	12 (48.0)	14 (56.0)	13 (52.0)	12 (48.0)	10 (40.0)	8 (32.0)	10 (40.0)	12 (48.0)	13 (52.0)	12 (48.0)	3 (50.0)	
ASA classification[b]														0.916
Ⅰ- Ⅱ	179 (63.7)	14 (56.0)	12 (48.0)	17 (68.0)	18 (72.0)	16 (64.0)	14 (56.0)	19 (76.0)	18 (72.0)	17 (68.0)	16 (64.0)	14 (56.0)	4 (66.7)	
Ⅲ	91 (32.4)	10 (40.0)	12 (48.0)	7 (28.0)	7 (28.0)	9 (36.0)	10 (40.0)	5 (20.0)	5 (20.0)	7 (28.0)	7 (28.0)	10 (40.0)	2 (33.3)	
Ⅳ	11 (3.9)	1 (4.0)	1 (4.0)	1 (4.0)	0 (0.0)	0 (0.0)	1 (4.0)	1 (4.0)	2 (8.0)	1 (4.0)	2 (8.0)	1 (4.0)	0 (0.0)	
AO/OTA classification[c]														0.676
A1	143 (50.9)	12 (48.0)	13 (52.0)	17 (68.0)	10 (40.0)	10 (40.0)	11 (44.0)	13 (52.0)	15 (60.0)	16 (64.0)	9 (36.0)	13 (52.0)	4 (66.7)	
A2	119 (42.3)	11 (44.0)	11 (44.0)	6 (24.0)	12 (48.0)	13 (52.0)	14 (56.0)	11 (44.0)	9 (36.0)	8 (32.0)	14 (56.0)	9 (36.0)	1 (16.7)	
A3	19 (6.8)	2 (8.0)	1 (4.0)	2 (8.0)	3 (12.0)	2 (8.0)	0 (0.0)	1 (4.0)	1 (4.0)	1 (4.0)	2 (8.0)	3 (12.0)	1 (16.7)	
Time to operation (hours)														0.913
≤24	275 (97.9)	24 (96.0)	24 (96.0)	25 (100.0)	25 (100.0)	24 (96.0)	24 (96.0)	24 (96.0)	25 (100.0)	25 (100.0)	24 (96.0)	25 (100.0)	6 (100.0)	
>24	6 (2.1)	1 (4.0)	1 (4.0)	0 (0.0)	0 (0.0)	1 (4.0)	1 (4.0)	1 (4.0)	0 (0.0)	0 (0.0)	1 (4.0)	0 (0.0)	0 (0.0)	

Abbreviation: BMI, body mass index (calculated as weight in kilograms divided by height in maters squared); ASA, American Society of Anesthesiologists; AO/OTA, the AO Foundation/Orthopaedic Trauma Association.

[a] Data are shown as number (percentage) if the variable is categorical and as mean ± standard deviation if continuous and normally distributed.

[b] Range, Ⅰ to Ⅳ; higher level indicates higher risk of anesthesia.

[c] Range, A1 to A3; different classification indicates different type of fracture. A1, simple fracture; A2, comminuted fracture involving lateral cortex; A3, reverse oblique fracture.

© 2020 Jia X et al. *JAMA Network Open*.

eFigure 1. Risk of All-Cause 90-Day Mortality in Propensity Score–Matched Patients

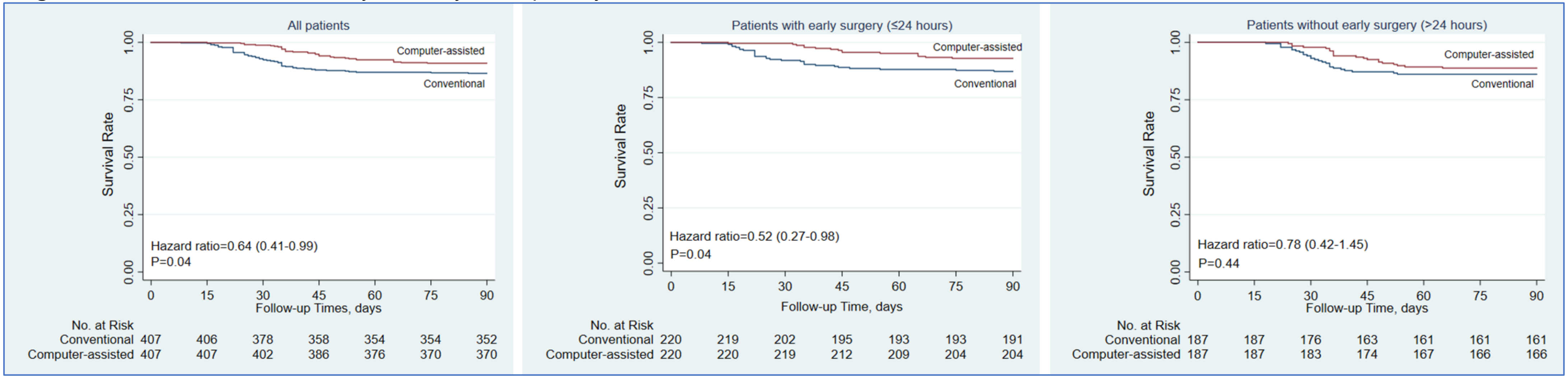

The hazard ratios indicate the risk of mortality in the computer-assisted group as compared with the conventional group; 95 percent confidence intervals are shown in parentheses. P values were calculated with use of the Cox proportional hazards models.

© 2020 Jia X et al. *JAMA Network Open*.

eFigure 2. Cumulative Incidence of All-Cause 90-Day Complications in Propensity Score–Matched Patients

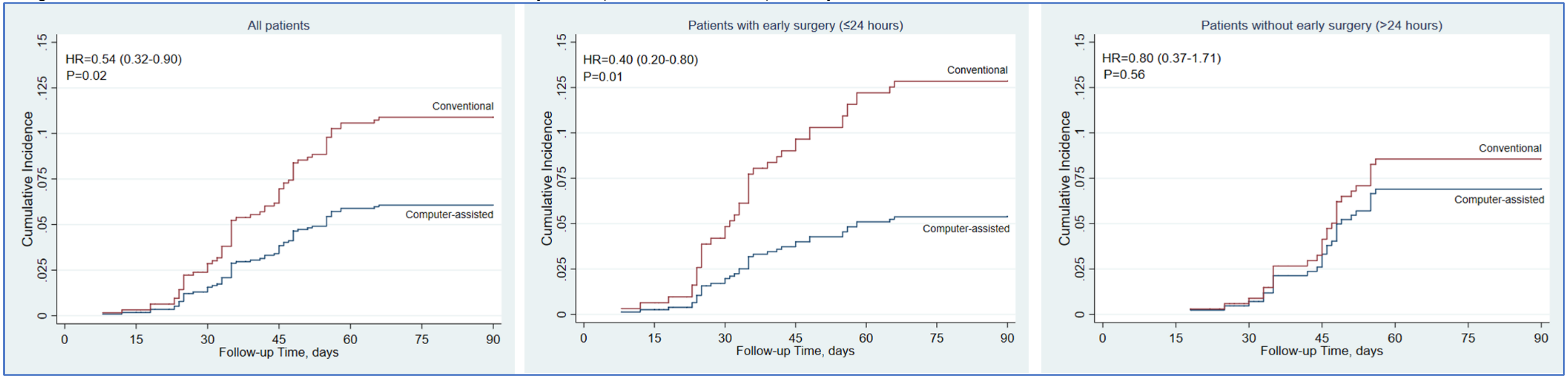

The hazard ratios indicate the cumulative incidence of complication in the computer-assisted group as compared with the conventional group; 95 percent confidence intervals are shown in parentheses. P values were calculated with use of the Gray test.

© 2020 Jia X et al. *JAMA Network Open*.

eFigure 3. Learning Curves of Closed Reduction of Fracture Time With Use of Computer-Assisted and Conventional Preoperative Planning

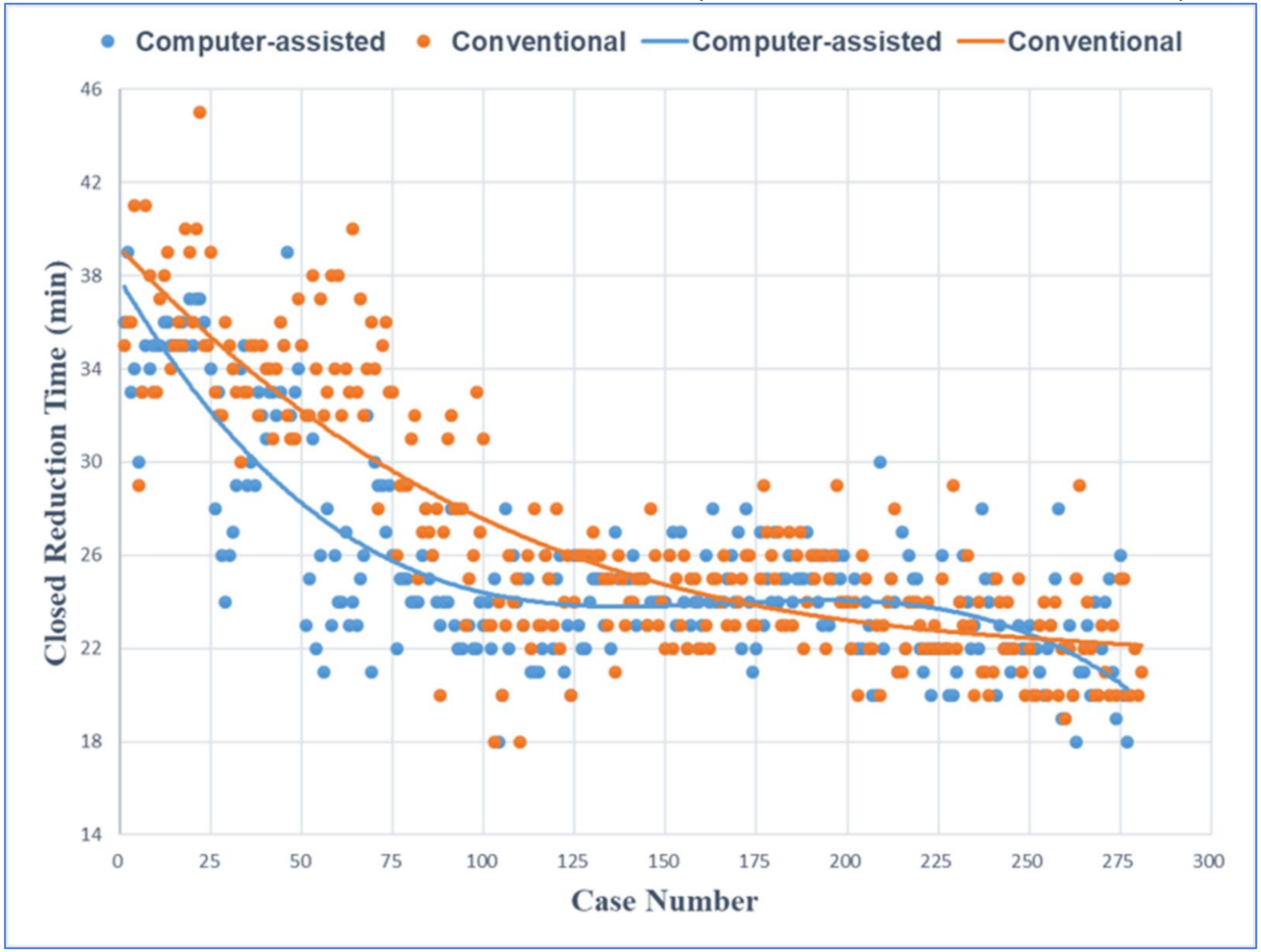

Computer-assisted: $y = -3E-06x^3 + 0.0016x^2 - 0.2653x + 37.805$, $R^2 = 0.6656$, $P < .001$; Conventional: $y = -7E-07x^3 + 0.0006x^2 - 0.167x + 39.185$, $R^2 = 0.7387$, $P < .001$.

© 2020 Jia X et al. *JAMA Network Open*.

eFigure 4. Learning Curves of Number of Fluoroscopies With Use of Computer-Assisted and Conventional Preoperative Planning

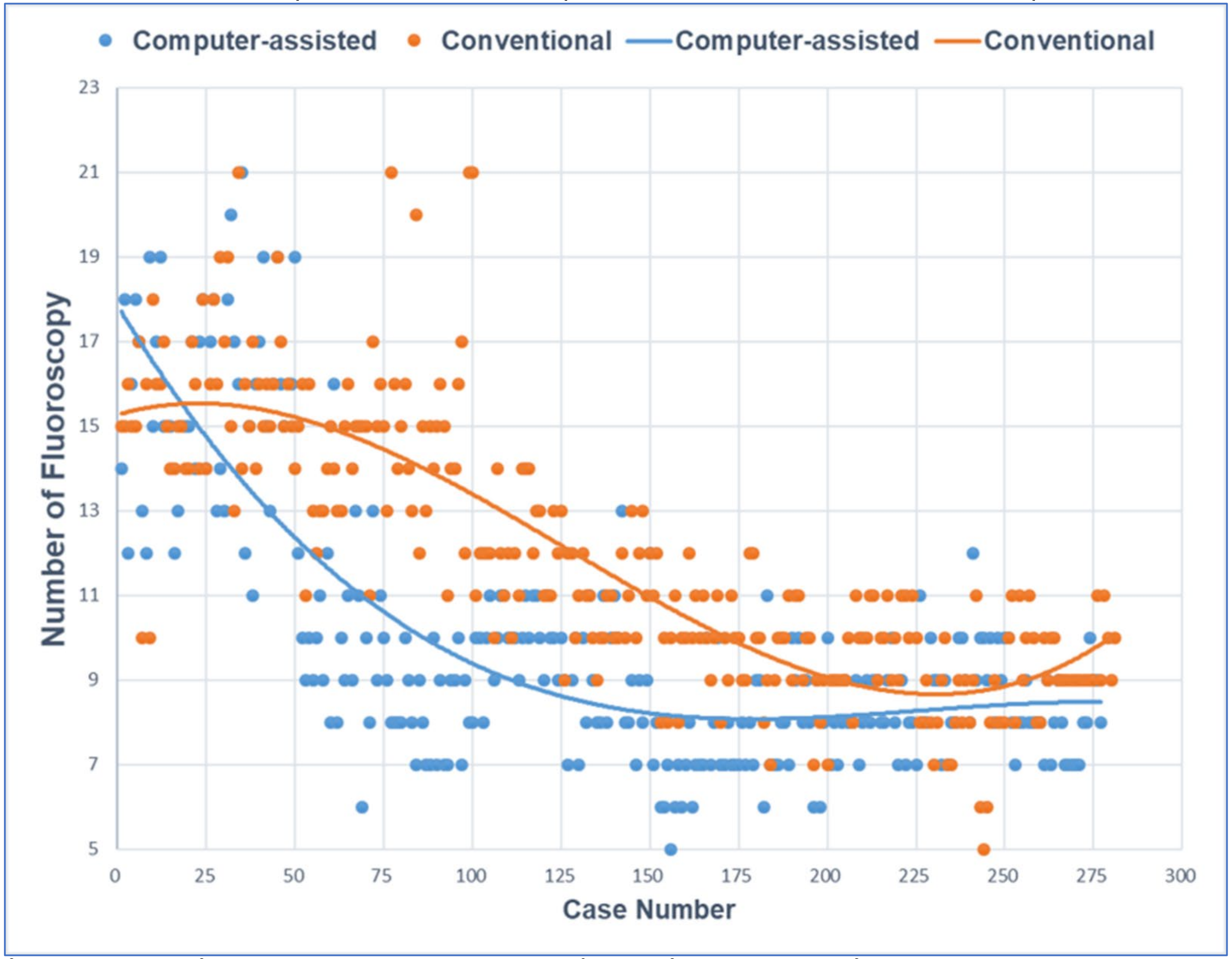

Computer-assisted: y = -9E-07x^3 + 0.0006x^2 - 0.1395x + 17.847, R^2 = 0.6366, P < .001; Conventional: y = 2E-06x^3 - 0.0006x^2 + 0.0241x + 15.27, R^2 = 0.6957, P < .001.

© 2020 Jia X et al. *JAMA Network Open*.

eFigure 5. Learning Curves of Estimated Blood Loss With Use of Computer-Assisted and Conventional Preoperative Planning

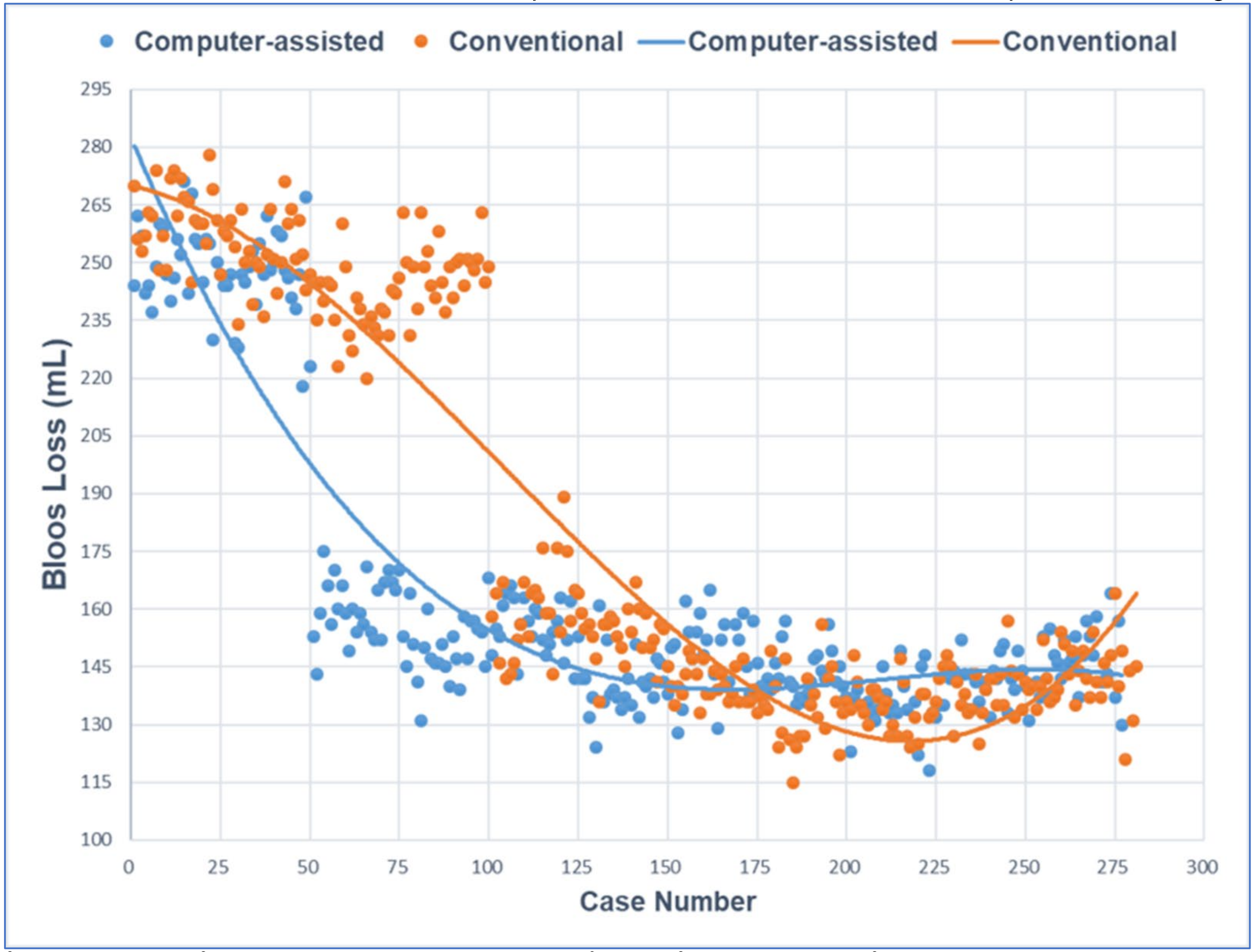

Computer-assisted: $y = -2E\text{-}05x^3 + 0.0109x^2 - 2.1925x + 282.49$, $R^2 = 0.8331$, $P < .001$; Conventional: $y = 2E\text{-}05x^3 - 0.0073x^2 - 0.2029x + 270.03$, $R^2 = 0.881$, $P < .001$.

© 2020 Jia X et al. *JAMA Network Open*.
